# Supplementary material for: Evaluating Efficacy of a COVID-19 Alternative Care Site Preparedness Assessment Tool for Catastrophic Healthcare Surge Capacity during Pandemic Response
Source: Healthcare (Basel). 2023 Jan 21;11(3):324. doi: 10.3390/healthcare11030324 (PMC9914666; doi:10.3390/healthcare11030324)
Supplement: Supplementary file 1 [file healthcare-11-00324-s001.zip › healthcare-2065691-supplementary.pdf]

The American Institute of Architects

Coronavirus Disease 2019 (COVID-19) Alternative Care Sites (ACS)

Preparedness Assessment Tool V2.0

This preparedness assessment tool contains information synthesized from non-crisis situations (design principles, available supporting evidence, or translation of applicable standards) in combination with federal documents issued during the COVID-19 crisis. The content was prepared with joint professional input from trained and experienced health care architects, designers, engineers, scientists, life-safety consultants, healthcare professionals, and hospital facility operations.

All US states and territories should be prepared for the arrival of patients with COVID-19. All hospitals and public health agencies need to ensure that ACS, both on a health care campus and in community settings, are capable of handling patient care during a pandemic public health response to:

- Maximize preservation of life;
- Mitigate the risk of spreading of pathogens, including SARS-CoV-2 within the facility;
- Promptly identify patients requiring investigation and isolation and transition patient(s) to the correct facility based on health care organization or public health authority having jurisdiction (AHJ) determinations;
- Care for a larger number of patients with varying conditions in the context of an escalating outbreak/epidemic/pandemic; and
- Ensure monitoring and management of ongoing safety for 24/7 operations.
- Address ethnic diversity and cultural competency , as well as marginalized, rural, homeless, and mental/behavioral health populations

The checklist does not describe mandatory requirements; it does highlight important areas for evaluation by hospital and public health agencies in selecting ACS for the care and treatment of COVID-19 or surge-capacity patients. The goal is to identify appropriate rapid-adaptive reuse of existing built environments such as convention centers, sports arenas, community centers, hotels, dormitories, or other space for occupancy during the pandemic.

Disclaimer: This document is modeled after the Centers for Disease Control and Prevention Comprehensive Hospital Preparedness Checklist for Coronavirus Disease 2019 with adaptation for evaluation of alternative care sites (ACS). Additionally, this document was prepared during a seven-day rapid response period during the "Alert Phase" of a pandemic response (as defined by the WHO Pandemic Influenza Risk Management Global Influenza Programme). This assessment tool represents the work of the AIA COVID-19 Task Force to educate and inform architects, health care practitioners, public health professionals, and authorities having federal, state, and/or local jurisdiction within the emerging pandemic response. This material is not intended to replace existing applicable laws, regulations, or professional standards.

Go/no go building selection

If the building lacks any of these basic building components, then the current facility is unlikely to be appropriate for patient care operations during a pandemic since it cannot be rapidly altered.

Prior to finalizing site selection(s), all considerations should be reviewed for suitability to support care for the intended patient population.

	Yes	No
**Age:** The proposed site is a newer building, built (or renovated) under contemporary codes (less than 20 years old).	☐	☐
**Life-Safety:** Confirm building meets minimum construction type and egress requirements as allowed by the Authority Having Jurisdiction (AHJ).	☐	☐
**Adequate Floor Area:** There is enough space to accommodate all required patient care functions. (Evaluate using the considerations below.)	☐	☐
**Municipal Water Supply:** Water quality and pressure will support safe clinical operations. (Evaluate using the considerations below.)	☐	☐
**Power Outlets:** There are a sufficient number of grounded outlets for the safety of patients and staff. (Evaluate using the considerations below.)	☐	☐

1.0 General conditions of tool use

1.1 Baseline parameters

Completed/Agreed

1.1.1 Operating authority

- Confirm which authorities have jurisdiction (e.g., local hospitals/health systems; hospital systems with inter-operating agreements; federal, state, or local departments of health/public health; US DOD, FEMA, National Guard, and state militias; or other combinations) ☐
- Confirm whether authority may change over the course of the temporary occupation ☐

1.1.2 Key health administrative roles

- Establish an on-site health care operations leadership structure to govern and coordinate medical affairs and patient care operations, and properly support those operations ☐
- Identify and confirm remote support required ☐

1.1.3 Facility-specific administration

- Establish the facility-based administrative structure to monitor, maintain, or manage:
  - IT and communications ☐
  - supply chain ☐
  - fire-hazard potential ☐
  - operations of permanent and portable HVAC equipment ☐
  - medical gas (surgical air and oxygen) supplies ☐
  - infection prevention protocols ☐

- bio-hazardous waste ☐

- Develop procedures and manage evacuation/movement of patients and staff in emergency conditions ☐
- Create emergency egress maps and train occupants in facility-specific life-safety procedures ☐

1.1.4 Risk management

- Establish authority of the local operator to act in the best interest of patients under its care, assess patient risk, prioritize treatments, allocate/assign all local assets, and adjust course as needed ☐
- Conduct and keep up-to-date a facility-based Safety Risk Assessment (SRA) (inclusive of infection control, security, medication safety, infection prevention, patient handling, and the possibility of injury associated with behavioral/mental health) ☐
- Comply with federal, state, and local laws (e.g., Americans with Disabilities Act [ADA], Health Insurance Portability and Accountability Act [HIPAA], Occupational Safety and Health Administration [OSHA]) while ensuring patient life-safety. (Conditions may differ from strategies employed in non-pandemic care) ☐
- Confirm with AHJ and local fire department if a 24/7 fire watch is required and available due to existing building life safety/fire alarm deficiencies ☐

1.1.5 Professional support for ACS selection & development

- Engage licensed, trained, and experienced health care architects and engineers as well as those design professionals familiar with the alternative care building type under consideration ☐

1.2 Temporary assets

- Confirm whether the operator may also be required to provide temporary medical staff, an electrical generator, additional HVAC equipment, modular gas generation, toilet and shower facilities, staff housing, food, and supplies ☐

1.3 Evolving recommendations

- These recommendations support a rapid-response facility solution, evolving as conditions and precedents warrant ☐

2.0 Functional requirements

2.1 Concept for operations

Yes/Agreed

- Patient level of care is identified as:
  - ambulatory (capable of self-preservation) ☐

- acute (incapable of self-preservation) ☐
- critical (e.g., requiring life support/mechanical ventilation) ☐

- Patient diagnosis for treatment has been identified for:
  - COVID-19 positive (COVID) ☐
  - COVID-19 negative (non-COVID) ☐
  - Transitional cases (e.g., Asymptomatic, Persons under Investigation (PUI), post-discharge) ☐
- Develop a plan for where both diagnosis and treatment will occur ☐
- Develop a plan for providing care for unanticipated mental and behavioral patients (e.g., observation, safe design areas, transfers) ☐

2.2 On-site patient care

2.2.1 Site selection/location (see also 2.2.6, 2.3.1)

- Site is in close proximity to first responders ☐
- Site is convenient to a hospital ☐
- Confirm parking for staff, ambulances, delivery, and waste removal ☐

2.2.2 Isolate COVID-19 operations

- COVID-related operations can be separated from other activities in the facility ☐
- Establish one-way flow of patient, staff, and materials from clean to contaminated ☐
- Isolate staff support spaces from patient care spaces ☐
- Establish plan for transitioning patient care type during operation of ACS ☐

2.2.3 Patient spaces are sized to accommodate:

- A projected number of ______ occupants, with:
  - direct or remote patient monitoring ☐
  - patient privacy ☐
  - daylight allowed in patient space (windows in patient rooms preferred) ☐
  - patient toileting and bathing ☐
  - handwashing sink for staff ☐
  - space/connections for ventilators, IV poles, monitors, biohazard disposal, etc. ☐

- Space for storage cart and disposal bin (recycling) of immediately available PPE ☐
- Space for donning and doffing of staff PPE-related attire at patient care areas with room for buddy system to supervise (or via camera observations) ☐
- Secure area for patient belongings ☐

2.2.4 Nurse/clinician team station spaces need to accommodate:

- A physician and nursing documentation area ☐
- An area for nurse/clinical team communication ☐
- A cardiopulmonary resuscitation cart ☐
- Handwashing sink for staff ☐
- A POCT (point of care testing) alcove in care areas (e.g., glucometer machines, i-STAT, arterial blood gases) ☐
- Nurse call annunciator ☐
- Surveillance monitors ☐

2.2.5 Nursing support needs to include:

- Provide a medication prep room (secured) or a self-contained dispensing unit with adequate lighting ☐
- Nourishment area(s) with sink, prep counter, refrigerator, ice machine, and handwash station ☐
- Clean supply room(s) ☐
- Soiled materials holding room with a flushable sink and cleanable work counter ☐
- Clean linen storage room ☐
- Equipment and supply storage room ☐
- Environmental services room ☐
- Secured storage for staff items ☐
- Staff toilet and shower ☐
- Area for facility-wide/unit PPE storage (secured as needed) ☐
- Ventilator decontamination and repair ☐
- Break room or respite area with access to daylight (where possible) ☐
- On-call sleeping rooms ☐

2.2.6 Intake & discharge

- Establish space needed for security protocols ☐

- Provide a covered ambulance drop-off ☐
- Accommodation for patient transfer related to disease case or death ☐

2.2.7 Intake & discharge if used for walk-ins

- Separate walk-in/drive-through patient registration ☐
- Enclosed, covered, and/or drive-through triage area (e.g., for swabbing, and evaluation) ☐
- Separate patient exit, remote from the patient entry ☐

2.2.8 End of life

- Accommodate virtual family access (adequate Wi-Fi) ☐
- Provide area for end-of-life visitation (based on allowed policies and protocols) ☐
- Provide exterior window if available (if conscious) ☐

2.3 General support

2.3.1 On-site support needs to include:

- Site security ☐
- Separate designated staff entry and exit consistent with SRA ☐
- Receiving and logistics ☐
- Satellite pharmacy and related security ☐
- A STAT-lab (See also POC in 2.2.5 Nursing Support) ☐
- Morgue (mobile, if necessary) ☐
- Normal and biohazard waste management ☐
- Decontamination and clean workroom for ventilators ☐
- Food delivery and staging ☐

2.3.2 Off-site support

- If not provided onsite, confirm inter-organizational operating agreements for:
  - sterile processing ☐
  - linen processing ☐
  - general equipment and supply storage and logistics, general lab ☐
  - general pharmacy ☐
  - lab ☐
  - food service ☐

2.3.3 Administration

- Areas to support site leadership

2.3.4 Caregiver support area for rest, self-isolation/ quarantine conditions

- Confirm location(s) for on-site temporary housing (medical, nursing, and support staff)
- Investigate ability to contract with local hotels or others providing sleeping quarters in close proximity

3.0 Facility modifications required under design/build

3.1 Architectural

3.1.1 Construction type

- Avoid combustible construction
- Preferred: NFPA 101, Life Safety Code Type II or greater level of fire and life-safety occupant protection

3.1.2 Passive life-safety

- Confirm if stairs allow for evacuation of patients on a med-sled, if patients are incapable of self-preservation
- Confirm egress paths for appropriate fire and smoke enclosure rating
- Review fire and smoke compartmentation based on patient density (especially if no or limited smoke dampers exist)
- Confirm general storage rooms >100 SF and oxygen storage rooms are fire-rated, including positive latching
- Confirm at least one horizontal exit
- Provide at least two means of egress per floor where patients are located
- Adequate exit signs and egress lighting

3.1.3 Defend in place

- Where patients are incapable of self-preservation, evaluate life-safety plans to defend in place or develop an active operationally-based life safety plan

3.1.4 Building geometry

- Confirm layout (walls, doors, corridors, etc.) for the ability of staff to easily move patient stretchers and medical equipment

- Confirm visibility for staff to maintain sightlines in patient care areas to actively monitor patients as well as mitigate risk of behavioral incidents (e.g., elopement, aggression—in the event of behavioral/mental health comorbidity) ☐

3.1.5 Building components

- Provide patient entries/doors that:
  - Include vision panels for out-of-room observation ☐
  - Contain aerosolized spores ☐
  - Allow clearances for equipment and stretcher ingress and egress ☐
- Provide scrubbable and easily cleaned finishes in patient areas (evaluate removal of existing finishes such wall coverings, window treatments, and carpet) ☐
- Provide hard-surface, slip-resistant flooring ☐

3.1.6 Equipment & furnishings

- Use patient beds and chairs that can withstand disinfection ☐
- Evaluate space for necessary furniture and medical equipment ☐
- Remove equipment or furniture not directly supporting patient care ☐

3.1.7 Conveyance

- Elevators should allow staff to move a patient on a stretcher or, if ambulatory, in a wheelchair ☐
- Consider segregating staff and patient flow in elevators ☐

3.2 Structural

- Confirm the existing structural system can support the revised structural (live) load for increased occupants, medical equipment, and any additional rooftop equipment ☐

3.3 Mechanical

3.3.1 Isolation & negative pressure

- If clinically required, make provisions to maintain a negative pressure room, zone, or floor ☐
- If possible and clinically required, use/create airborne infection isolation rooms (AIIR) ☐
- Where possible, locate exhaust near the patient’s head ☐
- Ensure adjacent spaces are protected from cross-contamination ☐

3.3.2 Air-changes & filtration

- Where possible, the preference is 10 air-changes per hour (ACH), plus 2 ACH of outside air ☐
- Where possible, the preference is MERV 7 filters at patient areas and MERV 14 final filters ☐

3.3.3 Testing & balancing

- Perform post-construction/renovation testing and balancing of the HVAC system ☐

3.3.4 Potential existing system limitations

- Consider through-wall units and bathroom exhaust for existing exhaust capacity, filtration, and air change rates. (These may be affected by ductwork sizing) ☐
- Consider packaged rooftop units with electric heating to provide additional ventilation ☐
- Consider upgrading building exhaust systems (e.g., higher-volume fans) to maintain negative pressure ☐
- Evaluate humidity controls to provide optimal humidity levels for effective patient care and treatment ☐
- Consider HEPA-filtered recirculation units in patient rooms ☐

3.3.5 Smoke control

- Confirm smoke-control system capabilities. If not possible, the patient population may be limited ☐

3.4 Electrical

3.4.1 Power

- Provide power circuits based on equipment demands at all patient and staff locations ☐
- Confirm locations of floor boxes for general power distribution ☐

3.4.2 Branch power availability

- Confirm branch circuits are available to support patient care operations ☐

3.4.3 Emergency power

- Based on the needs of the patients (i.e., ambulatory or critical care), provide uninterrupted critical and life-safety power circuits to support patient care areas for at least 24 hours for essential services, including:
  - life-sustaining medical equipment and drug storage equipment/refrigerators ☐

- outlets to support medical equipment at patient care stations and headwalls ☐
- nurse call system ☐
- IT (patient-related, medical team communication, and infrastructure) ☐
- egress lighting ☐
- fire alarm system, elevators, IT infrastructure, etc. ☐
- elevators ☐
- automatic transfer switch ☐

3.4.4 Lighting

- Provide lighting (sealed fixtures) and portable exam lights to support the clinical operation ☐
- Verify emergency egress lighting ☐

3.5 Plumbing/medical gas

3.5.1 Medical gas

- Provide accommodations for oxygen, medical air, and vacuum to support intubation and ventilator operations s to accommodate high/full demand ☐
- Confirm availability of space for temporary modular oxygen tank generators, when needed ☐
- Confirm availability of an appropriately fire-rated storage container or room for bottled oxygen ☐
- Confirm availability of storage for empty oxygen tanks ☐

3.5.2 Water

- Provide potable water to support the clinical operation. (This may include disinfection of the water main and the water distribution system and analytical testing of the building to confirm the water system is safe for patient care operations) ☐
- If required, plan for processing water for decontamination or on-site sterilization. ☐
- Plan for ongoing management of the water system to include flushing, temperature monitoring, and residual disinfectant levels. ☐

3.6 Fire protection/life safety

- Confirm fire/life safety conditions and expectations with local AHJ(s) ☐

- Confirm presence of fire alarm system and functionality to support the patient care setting. If no fire alarm system, reevaluate appropriateness of building for pandemic response, or install a fire alarm system ☐
- Preference: Full automatic fire-suppression system ☐
- Confirm adequate supply and spacing of fire extinguishers ☐

3.7 Communication

3.7.1 Information technology

- Confirm or install a secure information technology infrastructure appropriate to support patient care, including HIPAA compliance ☐

3.7.2 EMS communications

- Confirm or install an EMS communication system appropriate to support patient care, including HIPAA compliant security ☐

3.7.3 Nurse call/communication

- Confirm or install a secure nurse call system appropriate to support patient care or similar method of communication from patient to nurse. ☐
- Establish method for clinician communication (e.g., mobile, wireless) ☐

3.7.4 Fire alarm

- Confirm or install a fire alarm system connected to local emergency responders ☐
- Verify availability of fire alarm pull stations ☐

3.7.5 Security surveillance

- Evaluate security needs and provide security surveillance system as appropriate (cameras, monitors, motion detectors, door and window alarms etc.) ☐
- Consider wireless systems where necessary ☐

4.0 Additional considerations

4.1 Ethnically diverse or marginalized populations

- Review any space needs to address cultural competency ☐
- Consider accommodations for community-specific spiritual and cultural practices ☐
- Consider accessibility of ACS and transportation options and within or adjacent to public housing communities ☐
- Provide dedicated spaces for social worker(s) and language translation services ☐

4.2 Rural populations

- Consider accessibility of and navigation to ACS, evaluating associated drive times and distances ☐
- Consider the need for helicopter transport ☐
- Consider housing and lodging for healthcare staff and patient families ☐
- Evaluate needs for ambulance services to transport higher acuity patients ☐
- Verify systems for communication (mobile phone, boosters for improved wireless) ☐
- Verify critical infrastructure needs (sewer, septic, potable water, electricity) are adequate for anticipated demand ☐

4.3 Homeless and unsheltered populations

- Provide bathroom and shower areas, located within close proximity to entry ☐
- Designated storage for life belongings, bicycles etc. ☐

4.4 Mental and behavioral health (MBH) patients

- Consistent with section 1.1.4, perform a Safety Risk Assessment of all locations accessible to potential MBH patients to eliminate opportunities for self-harm and harm against others ☐
- If MBH patients are identified, provide space for continuous 1:1 observation until transfer or discharge ☐
- Consider safe rooms, if appropriate to operational plan identified ☐

Additional guidance

For additional reference materials, refer to https://bit.ly/C19HealthcareArch

Task force & author contributions

Members of the task force

(MS) Molly M. Scanlon, PhD, FAIA, FACHA – Chair
(DA) Diana Anderson, MD, ACHA
(EE) Eve A. Edelstein, PhD, MArch, Assoc. AIA, EDAC, FAAA
(JF) John Fowler, AIA, NCARB, EDAC, LEED AP
(WH) William Hercules, FAIA, FACHA, FACHE
(EP) Erin Peavey, AIA, EDAC, LEED AP
(YS) Yiselle Santos, AIA, LSSYB, WELL AP, LEED AP
(ET) Ellen Taylor, PhD, AIA, MBA, EDAC
(KW) Kirsten Waltz, AIA, ACHA, EDAC, LEED AP
(MW) Marvina Williams, RN, BSN, LSSBB
(FZ) Frank Zilm, DArch, FAIA, Emeritus FACHA
(DP) Doug Paul, AIA Staff

COVID-19 Alternative Care Sites Preparedness Assessment Tool
The following represents the individual author's substantial contributions: conceptualization, MS; formal codes and standards analysis, JF, WH, KW, and ET; project administration, ET, MS, and DP.

Meetings
The task force conducted daily meetings from March 24–April 2 2020 for the preparation of the Health Impact Brief #1 and related work products for review, consensus, and approvals. Additional subgroup meetings for each work product were held over the same time period. Updates were prepared through April 22, 2020 for a revised version release

Additional contributors to the COVID-19 Alternate Care Sites Preparedness Assessment Tool

Amanda Abney, Associate AIA, EDAC
Patricia Acevedo, AIA
Javan Arroyo
Joe Bonanno
Gabrielle Bullock, FAIA, NOMAC, IIDA, LEED AP
Daniel Caron, PE, LEED AP
Jason Costello, AIA, EDAC
Ashleigh Cotter, CID, CHID, IIDA, LEED AP BD+C, CLGB
Rachael Farrell, EDAC, LSSGB, LEED AP, BD+C
David Frum, FAIA, FACHA
Ruth Fanning, MD
Angel Gomez
Jeff Hankin, PE
Anita Honkanen, MD
Nathan Howell, AIA
Jennifer Burke Jackson, AIA, LEED AP
Isaac Karley
Ed Ledoux
Christopher Lynch, PE
Zach Mathern
Don MacAlister, CHPA
Todd Medd, AIA, NCARB
Christopher O'Brien, AIA, ACHA, EDAC, LEED AP
Sam Olbekson, AIA, NCARB, AICAE
John P. Riordan, MD
Nick Schumacher, PE
Michael Sheerin, PE, LEED AP
Hannah Shultz
Tom Smith, CHPA, CPP
Rick Taylor, RCDD
Kevin Tuohey, CHPA
Scott Waltz, RA
Julie Wellik
Tony York, CHPA, CPP

The American Institute of Architects
1735 New York Ave., NW
Washington, DC 20006
aia.org

Outil d'évaluation de l'état de préparation V2.0

Le présent outil d'évaluation de l'état de préparation contient des informations synthétisées provenant de documents rédigés pour des situations non urgentes (principe de conception, preuves justificatives disponibles ou traductions des normes applicables) couplés à des documents fédéraux émis au cours de la crise de la COVID-19. Le contenu a été préparé sur la base des contributions professionnelles d'architectes, de concepteurs, d'ingénieurs, de scientifiques expérimentés et formés au secteur de la santé, de consultants spécialisés dans la sécurité des personnes, de professionnels de la santé et d'observations tirées des activités hospitalières.

Tous les États et territoires des États-Unis doivent être préparés à l'arrivée de patients atteints par la COVID-19. Les hôpitaux et les organismes de santé publique doivent s'assurer que les ACS, que ce soit dans un établissement de soins de santé ou dans les établissements des collectivités, sont en mesure de prendre en charge des patients dans le cadre d'une réponse de santé publique à une pandémie aux fins de :

- Préserver au mieux les vies humaines ;
- Réduire le risque de propagation des agents pathogènes, y compris le SARS-CoV-2, dans l'établissement ;
- Promptement identifier les patients devant être examinés et isolés et transférer les patients dans les établissements appropriés sur la base des décisions des autorités sanitaires ou des organisations de soins de santé compétentes (AC) ;
- Prendre soin d'un grand nombre de patients infectés à des degrés variables dans le contexte d'une éclosion/épidémie/pandémie ;
- Assurer le suivi et la gestion de la sécurité permanente des opérations 24/7 ; et
- Assurer des compétences culturelles et multiethniques et répondre aux besoins des populations marginalisées, rurales, itinérantes et souffrant de problèmes de santé mentale ou comportementale.

La liste de contrôle ne contient pas de dispositions obligatoires ; elle souligne les domaines importants devant faire l'objet d'une évaluation par les hôpitaux et les organismes de santé publique qui sont amenés à sélectionner des ACS pour accueillir et soigner les patients infectés par la COVID-19 ou en cas de forte augmentation du nombre de patients. L'objectif est d'identifier la réutilisation rapide et adaptive d'établissements existants, comme les centres de conventions, les arènes de sport, les centres communautaires, les hôtels, les dortoirs et autres espaces pouvant être occupés au cours de la pandémie.

Avis de non-responsabilité : Le présent document s'inspire du modèle de la Liste exhaustive de contrôle de préparation des hôpitaux à la maladie à coronavirus 2019 des Centres pour le contrôle et la prévention des maladies (CDC, Centers for Disease Control and Prevention), adapté à l'évaluation des sites de soins alternatifs (ACS, Alternative Care Sites). Ce document a été préparé au cours d'une période d'intervention rapide de sept jours au cours de la « Phase d'alerte » du plan de réponse à une pandémie (telle que définie par le programme global de gestion des risques de pandémie de grippe de l'OMS). Le groupe d'études AIA COVID-19 a élaboré cet outil d'évaluation aux fins d'éduquer et d'informer les architectes, les praticiens de la santé, les professionnels de la santé publique et les autorités habilitées à intervenir au niveau local, étatique et/ou fédéral dans la lutte contre la pandémie émergente. Ce texte n'a pas vocation à remplacer les lois, règlements et normes professionnelles en vigueur.

À faire/Ne pas faire pour sélectionner un bâtiment

Si l'un des éléments fondamentaux de construction suivants n'est pas présent dans le bâtiment, il est peu probable que l'établissement soit adapté à des activités de soins des patients au cours d'une pandémie puisqu'il ne sera pas possible de les modifier rapidement.

Avant de procéder au choix définitif du site, tous les éléments d'appréciation doivent être examinés pour déterminer l'aptitude du site à accueillir des services de soins de santé pour les groupes de patients visés.

	Oui	Non
**Âge :** Le site proposé est une construction relativement récente, construite (ou rénovée) en conformité aux normes actuelles (moins de 20 ans d'âge).	○	○
**Sécurité des personnes :** Confirmer que le bâtiment respecte les conditions minimales relatives au type de construction et aux évacuations, telles que déterminées par les autorités compétentes (AC).	○	○
**Surface de plancher adéquate :** Il doit y avoir assez d'espace pour accueillir toutes les fonctions de soins des patients. (Évaluer en tenant compte des éléments d'appréciation ci-après.)	○	○
**Alimentation municipale en eau :** La qualité et la pression de l'eau assureront la sécurité des opérations cliniques. (Évaluer en tenant compte des éléments d'appréciation ci-après.)	○	○
**Prises d'alimentation électrique :** Il y a un nombre suffisant de prises électriques reliées à la terre pour assurer la sécurité des patients et du personnel. (Évaluer en tenant compte des éléments d'appréciation ci-après.)	○	○

1.0 Conditions générales d'utilisation des outils

1.1 Paramètres de référence

Achevé/approuvé

1.1.1 Autorité responsable

- Confirmer qui sont les autorités compétentes (p. ex. les hôpitaux/systèmes de santé locaux, les organes hospitaliers ayant passé des accords opérationnels, les services de santé/santé publique locaux, étatiques ou fédéraux, DOD (département de la Défense des États-Unis), FEMA (*Federal Emergency Management Agency*, Agence fédérale de gestion des situations d'urgence), la Garde nationale et les milices d'État, ou toute autre combinaison) ☐
- Indiquer si l'autorité responsable est susceptible de changer au cours de l'occupation temporaire ☐

1.1.2 Fonctions administratives essentielles de santé publique

- Établir in situ une structure dirigeante des opérations sanitaires qui gérera et coordonnera les questions médicales et les soins administrés aux patients ; soutenir adéquatement ces opérations ☐
- Identifier et confirmer l'assistance à distance requise ☐

1.1.3 Administration de l'établissement

- Instaurer une structure administrative dans l'établissement qui surveillera, maintiendra ou gérera ce qui suit :

- IT et communications ☐
- chaîne logistique ☐
- risque d'incendie ☐
- tâches liées à l'équipement CVC permanent et portable ☐
- approvisionnement en gaz médicaux (air et oxygène chirurgicaux) ☐
- protocoles pour la prévention des infections ☐
- déchets biomédicaux ☐

• Développer des procédures d'évacuation/de circulation des patients et du personnel en cas d'urgence et savoir les gérer ☐

• Créer un plan des sorties de secours et former les occupants aux procédures de sécurité des personnes et du bâtiment ☐

1.1.4 Gestion des risques

• Habiliter l'exploitant local à agir dans l'intérêt supérieur des patients qui lui sont confiés, à évaluer le risque des patients, à hiérarchiser les traitements, à allouer/affecter les actifs sur place et ajuster en fonction des besoins ☐

• Effectuer et mettre à jour une évaluation des risques de sécurité (ERS) de l'établissement (y compris du contrôle de l'infection, de la sécurité, de la sûreté des médicaments, de la prévention de l'infection, du traitement des patients et des risques potentiels de blessures liées aux troubles de santé mentale et comportementale) ☐

• Se conformer aux lois locales, étatiques et fédérales (par exemple, la loi relative aux Américains en situation de handicap (*Americans with Disabilities Act*, ADA), la loi sur la portabilité et la responsabilité en assurance santé (*Health Insurance Portability and Accountability Act*, HIPAA), la loi sur la sécurité et la santé au travail (*Occupational Safety and Health Administration*, OSHA)) tout en assurant la sécurité des patients. (Les conditions peuvent différer des stratégies de soins utilisées hors du contexte d'une pandémie) ☐

• Confirmer avec les AC et la caserne de pompiers locale si une surveillance 24/7 est requise et disponible en raison des déficiences des systèmes d'alarme incendie et de sécurité du bâtiment ☐

1.1.5 Soutien professionnel pour la sélection et le développement des ACS

• Embaucher des architectes et des ingénieurs agréés, expérimentés et formés au secteur de la santé ainsi que des concepteurs professionnels connaissant le type de bâtiments alternatifs envisagés ☐

1.2 Actifs temporaires

- Confirmer si l’exploitant peut aussi être tenu de fournir du personnel médical temporaire, un générateur électrique, de l’équipement CVC supplémentaire, un générateur de gaz modulaire, des toilettes et des douches, des logements, de la nourriture et des fournitures pour le personnel ☐

1.3 Recommandations évolutives

- Ces recommandations visent à appuyer une force de réponse rapide, qui évoluera lorsque les conditions et les précédents l’exigeront ☐

2.0 Exigences fonctionnelles

2.1 Schéma opérationnel

Oui/Approuvé

- Le niveau de soins fournis aux patients est identifié comme suit :
  - ambulatoire (capable d’assurer son autoprotection) ☐
  - aigu (incapable d’assurer son autoprotection) ☐
  - critique (p. ex., requérant un ventilateur mécanique ou de réanimation) ☐
- Le diagnostic des patients nécessitant un traitement a été identifié pour :
  - Testé positif à la COVID-19 (COVID) ☐
  - Testé négatif à la COVID-19 (COVID) ☐
  - Cas transitoires (p. ex., asymptomatiques, personnes placées en observation, sortie de l’hôpital) ☐
- Développer un plan visant à la fois le diagnostic et le traitement ☐
- Développer un protocole de soins pour des patients aux prises avec des problèmes imprévus de santé mentale et comportementale (p. ex. observation, zones de sécurité, transferts) ☐

2.2 Soins administrés in situ aux patients

2.2.1 Sélection/lieu du site (voir aussi 2.2.6, 2.3.1)

- Site est à proximité des premiers secours ☐
- Site est situé près d’un hôpital ☐
- Confirmer le parking pour le personnel, les ambulances, les livraisons et l’enlèvement des déchets ☐

2.2.2 Isoler les opérations liées à la COVID-19

- Les opérations liées à la COVID peuvent être séparées des autres activités au sein de l'établissement ☐
- Instaurer une circulation à sens unique pour les patients, le personnel et le matériel qui va de propre à contaminé ☐
- Isoler les zones de travail dédiées au personnel des zones dédiées aux soins des patients ☐
- Établir un plan pour les soins des patients en transition au cours du fonctionnement des ACS ☐

2.2.3 La taille des zones dédiées aux patients convient à :

- Un nombre d'occupants estimé à ______ avec :
  - surveillance directe ou à distance des patients ☐
  - vie privée des patients ☐
  - lumière du jour admise dans l'espace du patient (préférence pour des fenêtres dans les chambres des patients) ☐
  - toilettes et bain pour les patients ☐
  - lavabo pour le lavage des mains du personnel ☐
  - espace/connexions pour les ventilateurs, tiges porte-sérum, écrans, enlèvement des déchets biomédicaux, etc. ☐
- Espace pour le chariot de rangement et poubelles (recyclage) de l'ÉPI immédiatement disponible ☐
- Espace pour mettre et enlever l'ÉPI du personnel dans les zones de soins des patients avec de la place pour un système de surveillance mutuelle (ou surveillance par caméra) ☐
- Zone sécurisée pour les affaires personnelles des patients ☐

2.2.4 La station des infirmières/équipe clinique doit être assez large pour accommoder :

- Une zone de documentation des patients pour les médecins et les infirmières ☐
- Un espace pour la communication infirmière/équipe clinique ☐
- Un chariot de réanimation cardiorespiratoire ☐
- Un lavabo pour le lavage des mains du personnel ☐
- Un point de tests au patient (POCT, *point of care testing*) dans les zones de soins (p. ex., analyseur glucose, i-STAT, gaz du sang artériel) ☐
- Un système d'appel des infirmières ☐
- Des écrans de surveillance ☐

2.2.5 Pour répondre aux besoins des infirmières, prévoir :

- un espace de préparation des médicaments (sécurisé) ou un distributeur autonome suffisamment éclairé ☐
- une/des zone(s) d'alimentation avec un évier, un comptoir de préparation, un réfrigérateur, une machine à glaçons et une station pour se laver les mains ☐
- une/des salle(s) pour entreposer le matériel propre ☐
- une salle pour entreposer le matériel souillé avec évier rinçable et comptoir nettoyable ☐
- une salle pour entreposer le linge propre ☐
- une salle pour entreposer l'équipement et les fournitures ☐
- une salle pour les services environnementaux ☐
- une salle de dépôt des effets du personnel ☐
- des toilettes et douche pour le personnel ☐
- une aire pour entreposer les ÉPI du service/de l'établissement (sécurisée selon les besoins) ☐
- un service de décontamination et de réparation des ventilateurs ☐
- une aire de repos ou de pause avec accès à la lumière naturelle (si possible) ☐
- des chambres de repos pour les gardes ☐

2.2.6 Admission et sortie

- Évaluer l'espace requis pour les protocoles de sécurité ☐
- Fournir une aire couverte d'arrivée des ambulances ☐
- Prendre des dispositions pour le transfert des patients en cas de maladie ou de décès ☐

2.2.7 Admission et sortie des patients sans RDV

- Enregistrement séparé des patients ambulatoires ou arrivant en voiture ☐
- Zone de triage fermée, couverte et/ou d'arrivée en voiture (p. ex. pour l'écouvillonnage et l'évaluation) ☐
- Sortie distincte des patients, éloignée de la zone d'entrée des patients ☐

2.2.8 Fin de vie

- Prévoir l'accès virtuel des familles (wi-fi adéquat) ☐
- Aire de visite pour les patients en fin de vie (conformément aux protocoles et règlements autorisés) ☐
- Fournir une fenêtre extérieure si possible (si conscient) ☐

2.3 Assistance générale

2.3.1 L’assistance in situ inclut :

- Sécurité du site ☐
- Entrée et sortie désignées et dédiées du personnel conformes aux RSE ☐
- Livraisons et logistique ☐
- Pharmacie satellite et sécurité connexe ☐
- Un laboratoire STAT (technologies avancées et chirurgicales) (Voir aussi POCT au 2.2.4 Aide aux infirmières) ☐
- Morgue (mobile, si nécessaire) ☐
- Gestion des déchets ordinaires et biomédicaux ☐
- Zone de décontamination et de nettoyage des ventilateurs ☐
- Livraison et préparation de la nourriture ☐

2.3.2 Assistance hors site

- Si indisponible sur place, confirmer les accords opérationnels entre organisations pour :
  - stérilisation ☐
  - traitement du linge ☐
  - entreposage et logistique de l’équipement et des produits, laboratoire général ☐
  - pharmacie générale ☐
  - laboratoire ☐
  - service alimentaire ☐

2.3.3 Administration

- Zone de soutien à la direction ☐

2.3.4 Aire de repos pour le personnel soignant, conditions de quarantaine/auto-isolement

- Confirmer le ou les lieux d’hébergement temporaire sur site (personnel médical, infirmières et personnel de soutien) ☐
- Étudier la capacité à contracter avec des hôtels locaux ou autres lieux de logement à proximité ☐

3.0 Modifications requises pour concevoir/construire l’établissement

3.1 Architecture

3.1.1 Type de construction

- Éviter les matériaux de construction combustibles ☐
- De préférence : NFPA 101, code de sécurité de Type II ou niveau de protection supérieure pour la sécurité des occupants et contre les risques d’incendie ☐

3.1.2 Système de sécurité passive

- Confirmer si les escaliers permettent l’évacuation des patients sur une civière pour les patients ne pouvant s’autoprotéger ☐
- Confirmer les itinéraires de sortie pour l’indice de protection pertinent en cas de fumée et d’incendie ☐
- Examiner les cloisonnements antifeu ou antifumée en fonction de la densité des patients (surtout s’il n’y a pas ou peu de coupe-fumée) ☐
- Confirmer que les locaux d’entreposage >9 m^2 et que les salles de stockage d’oxygène sont résistantes au feu et équipées d’un mécanisme de verrouillage sûr ☐
- Confirmer au moins une issue de sortie horizontale ☐
- Prévoir au moins deux voies de sortie par étage accueillant des patients ☐
- Panneaux de sortie et éclairage des voies d’évacuation appropriés ☐

3.1.3 Défendre sur place

- Si les patients ne peuvent s’autoprotéger, évaluer les plans de sécurité des personne pour se protéger sur place ou développer un plan opérationnel pour la sécurité des personnes ☐

3.1.4 Géométrie du bâtiment

- Confirmer les plans au sol (murs, portes, couloirs etc.) pour que le personnel puisse facilement déplacer les brancards des patients et l’équipement médical ☐
- Confirmer la visibilité par le personnel du dispositif de signalisation dans les zones de soins des patients pour surveiller activement les patients et réduire les risques d’incident de type comportemental (fugue, agression en cas de comorbidité pour les troubles de santé mentale/comportementale) ☐

3.1.5 Composants des bâtiments

- Prévoir des portes/passages pour les patients qui :
  - incluent des panneaux transparents permettant l’observation hors chambre ☐

- contiennent des spores aérosolisées ☐
- permettent des dégagements pour l’entrée et la sortie de l’équipement et des brancards ☐

- Prévoir de l’équipement brossable et facilement nettoyable dans les zones des patients (évaluer l’enlèvement des finitions comme les revêtements des murs, les rideaux et la moquette) ☐
- Prévoir un revêtement de sol dur et antidérapant ☐

3.1.6 Équipement et meubles

- Utiliser des lits et des chaises pour patient résistants à la désinfection ☐
- Évaluer l’espace nécessaire pour les meubles et l’équipement médical ☐
- Enlever tout équipement ou meuble sans lien direct avec les soins au patient ☐

3.1.7 Moyens de transport

- Les ascenseurs doivent permettre au personnel de transporter un patient sur un brancard ou, pour les patients ambulatoires, dans un fauteuil roulant ☐
- Envisager des ascenseurs dédiés au personnel et aux patients ☐

3.2 Structure

- Confirmer que la structure existante peut accueillir une capacité de surcharge révisée en cas d’augmentation du nombre d’occupants, d’un surplus d’équipement médical et d’équipements sur toit supplémentaires ☐

3.3 Éléments mécaniques

3.3.1 Isolation et pression négative

- Si cliniquement requis, utiliser/créer des chambres d’isolement pour les infections à transmission aérienne (AIIR) ☐
- Si possible et si cliniquement requis, utiliser/créer des chambres d’isolement pour les infections à transmission aérienne (AIIR) ☐
- Si possible, localiser les sorties d’air au-dessus de la tête du patient ☐
- Assurer que les espaces adjacents sont protégés de toute contamination croisée ☐

3.3.2 Changement et filtration de l’air

- Si possible, la préférence va à un taux de 10 changements d’air par heure (ACH), plus 2 ACH d’air extérieur ☐

- Si possible, la préférence va aux filtres MERV 7 dans les zones des patients et aux filtres finaux MERV 14 ☐

3.3.3 Réglage et équilibrage

- Effectuer le réglage et l'équilibrage des systèmes de CVC après construction/rénovation ☐

3.3.4 Limite possible des systèmes existants

- Envisager des unités de ventilation murales et un ventilateur d'extraction dans les salles de bains pour le système de ventilation, la filtration et les taux de changement d'air existants (Tenir compte de la taille des conduits) ☐
- Envisager des unités à installer sur le toit avec chauffage électrique pour obtenir une aération supplémentaire ☐
- Envisager de remplacer les systèmes d'échappement existants (p. ex. ventilateurs à capacité supérieure) pour maintenir la pression négative ☐
- Évaluer le contrôle de l'humilité pour fournir des niveaux d'humidité optimale garantissant l'efficacité des soins et des traitements ☐
- Envisager des unités de recyclage de l'air avec filtres HEPA dans les chambres des patients ☐

3.3.5 Contrôle des fumées

- Confirmer la capacité du système de contrôle de fumées. En cas d'impossibilité, le nombre de patients doit être limité ☐

3.4 Alimentation électrique

3.4.1 Électricité

- Prévoir des circuits électriques tenant compte des exigences des équipements dans toutes les zones accueillant les patients et le personnel ☐
- Confirmer la localisation des boîtes de sol pour la distribution générale d'électricité ☐

3.4.2 Disponibilité des branchements

- Confirmer la disponibilité des circuits en dérivation pour les soins des patients ☐

3.4.3 Alimentation électrique de secours

- Selon les besoins des patients (ambulatoires ou soins critiques), fournir des circuits d'alimentation électrique de secours ou critiques sans interruption dans les zones de soins des patients pour alimenter au moins 24 heures de services essentiels, y compris :

- l'équipement médical de survie et la zone de stockage des médicaments et les réfrigérateurs ☐
- des prises pour l'équipement médical aux postes de soins des patients et prises murales ☐
- des systèmes d'appel infirmière ☐
- la TI (liée au patient, communication de l'équipe médicale et infrastructure) ☐
- l'éclairage des évacuations ☐
- le système d'alarme incendie, ascenseurs, infrastructure TI, etc. ☐
- les ascenseurs ☐
- l'inverseur de source automatique ☐

3.4.4 Éclairage

- Fournir de l'éclairage (appareils étanches) et des lampes d'examen médical portables pour les opérations cliniques ☐
- Vérifier l'éclairage des évacuations d'urgence ☐

3.5 Gas médical/plomberie

3.5.1 Gas médical

- Vérifier l'alimentation en oxygène, air médical et vide pour les activités d'intubation et de ventilation pour répondre à des demandes élevées ou à plein régime ☐
- Confirmer l'espace disponible pour stocker des générateurs d'oxygène modulaires temporaires et des bouteilles d'oxygène, en cas de besoin ☐
- Confirmer la disponibilité de salles d'entreposage ou de réservoirs de stockage résistants au feu pour les bouteilles d'oxygène ☐
- Confirmer un lieu d'entreposage des bouteilles d'oxygène vides ☐

3.5.2 Eau

- Fournir de l'eau potable pour les soins cliniques (Cela peut inclure la désinfection de la prise d'eau principale ou du système de distribution d'eau et des essais analytiques sur le bâtiment pour confirmer la compatibilité du système d'eau aux soins des patients) ☐
- Le cas échéant, prévoir un système de décontamination de l'eau ou de stérilisation sur place ☐
- Prévoir un système de contrôle permanent de l'eau incluant le rinçage, le contrôle de la température et les niveaux de désinfectants résiduels ☐

3.6 Sécurité-incendie et sécurité des personnes

- Confirmer les conditions et attentes en matière de sécurité-incendie et de sécurité des personnes avec les AC locales ☐
- Confirmer la présence de systèmes d'alarme incendie et leur fonctionnement pour protéger les espaces de soins des patients En cas d'absence d'alarme incendie, réévaluer la pertinence du bâtiment pour répondre à la pandémie ou installer un système d'alarme incendie ☐
- Préférence : système automatique d'extinction d'incendie ☐
- Confirmer la présence d'un nombre suffisant d'extincteurs d'incendie et l'espacement requis. ☐

3.7 Communication

3.7.1 Technologie de l'information

- Confirmer ou installer une infrastructure informatique sécurisée adaptée aux soins des patients et conforme à la norme de sécurité HIPAA ☐

3.7.2 Communications EMS

- Confirmer ou installer un système de communication adapté aux soins des patients et conforme à la norme de sécurité HIPAA ☐

3.7.3 Système d'appel infirmière/communication

- Confirmer ou installer un système d'appel infirmière adapté aux soins des patients ou une méthode de communication patient-infirmière similaire ☐
- Instaurer une méthode de communication pour les cliniciens (p. ex., mobile, sans fil) ☐

3.7.4 Alarme incendie

- Confirmer ou installer un système d'alarme incendie connecté aux services d'urgence locaux ☐
- Vérifier la disponibilité du dispositif de déclenchement d'alarme ☐

3.7.5 Surveillance de sécurité

- Évaluer les besoins en sécurité et fournir des systèmes adaptés de surveillance à des fins de sécurité (caméras, écrans, détecteurs de mouvement, alarmes porte et fenêtres ☐
- Envisager des systèmes sans fil si nécessaire ☐

4.0 Considérations supplémentaires

4.1 Populations marginalisées ou d'origines ethniques diverses

- Évaluer l'espace requis pour accueillir les compétences culturelles ☐
- Envisager un espace pour les pratiques culturelles et spirituelles propres à certaines communautés ☐
- Tenir compte de l'accès aux ACS, de l'offre de transport et de la proximité de logements sociaux ☐
- Prévoir un espace dédié aux travailleurs sociaux et aux services linguistiques ☐

4.2 Populations rurales

- Tenir compte de l'accès aux ACS, évaluer les distances géographiques et la durée des trajets ☐
- Tenir compte des besoins en transport par hélicoptère ☐
- Tenir compte du logement pour le personnel soignant et les familles des patients ☐
- Évaluer les besoins des services d'ambulance pour le transport des patients en état critique ☐
- Vérifier les systèmes de communication (téléphone portable, amplificateurs pour améliorer la connexion sans fil) ☐
- Vérifier si les infrastructures essentielles (égouts, fosses septiques, eau potable, électricité) ont une capacité suffisante pour répondre à la demande prévue ☐

4.3 Populations itinérantes et sans-abris

- Prévoir des douches et des toilettes situées à proximité de l'entrée ☐
- Espaces de rangement dédiés pour les effets personnels, les bicyclettes, etc. ☐

4.4 Patients souffrant de problèmes de santé mentale et comportementale

- Conformément à la section 1.1.4, mener une évaluation des risques de sécurité de tous les lieux accessibles aux patients souffrant d'éventuels troubles de santé mentale afin d'éliminer les risques d'automutilation ou de violence envers les tiers ☐
- Si des patients souffrant de santé mentale sont identifiés, prévoir un espace permettant une observation continue jusqu'au transfert ou à la sortie ☐
- Prévoir des salles sécurisées, si conforme au plan opérationnel identifié ☐

Orientation supplémentaire

Des documents de référence supplémentaires sont disponibles sur :
https://bit.ly/C19HealthcareArch

Contribution des auteurs et du groupe d'études

Membres du groupe d'études

(MS) Molly M. Scanlon, PhD, FAIA, FACHA – Chair
(DA) Diana Anderson, MD, ACHA
(EE) Eve A. Edelstein, PhD, MArch, Assoc. AIA, EDAC, FAAA
(JF) John Fowler, AIA, NCARB, EDAC, LEED AP
(WH) William Hercules, FAIA, FACHA, FACHE
(EP) Erin Peavey, AIA, EDAC, LEED AP
(YS) Yiselle Santos, AIA, LSSYB, WELL AP, LEED AP
(ET) Ellen Taylor, PhD, AIA, MBA, EDAC
(KW) Kirsten Waltz, AIA, ACHA, EDAC, LEED AP
(MW) Marvina Williams, RN, BSN, LSSBB
(FZ) Frank Zilm, DArch, FAIA, Emeritus FACHA
(DP) Doug Paul, AIA Staff

Outil d'évaluation de l'état de préparation des sites de soins alternatifs accueillant les malades du COVID-19
Les contributions principales de l'auteur sont les suivantes : conceptualisation, MS ; analyse des codes et normes officiels, JF, WH, KW et ET ; administration du projet, ET, MS et DP.

Réunions
Le groupe d'études s'est réuni quotidiennement du 24 mars au 2 avril 2020 pour la préparation du dossier n° 1 d'impact sur la santé et les documents connexes à des fins d'examen, de consensus et d'approbation. Des réunions en sous-groupes ont été organisées sur la même période pour les documents connexes. Des mises à jour ont été préparées jusqu'au 22 avril 2020 pour la publication d'une version révisée.

Autres contributeurs à l'outil d'évaluation de l'état de préparation des sites de soins alternatifs accueillant les malades du COVID-2019

Amanda Abney, Associate AIA, EDAC
Patricia Acevedo, AIA
Javan Arroyo
Joe Bonanno
Gabrielle Bullock, FAIA, NOMAC, IIDA, LEED AP
Daniel Caron, PE, LEED AP
Jason Costello, AIA, EDAC
Ashleigh Cotter, CID, CHID, IIDA, LEED AP BD+C, CLGB
Rachael Farrell, EDAC, LSSGB, LEED AP, BD+C
David Frum, FAIA, FACHA
Ruth Fanning, MD
Angel Gomez
Jeff Hankin, PE
Anita Honkanen, MD
Nathan Howell, AIA
Jennifer Burke Jackson, AIA, LEED AP
Isaac Karley
Ed Ledoux
Christopher Lynch, PE
Zach Mathern
Don MacAlister, CHPA
Todd Medd, AIA, NCARB
Christopher O'Brien, AIA, ACHA, EDAC, LEED AP
Sam Olbekson, AIA, NCARB, AICAE
John P. Riordan, MD
Nick Schumacher, PE
Michael Sheerin, PE, LEED AP
Hannah Shultz
Tom Smith, CHPA, CPP
Rick Taylor, RCDD
Kevin Tuohey, CHPA
Scott Waltz, RA
Julie Wellik
Tony York, CHPA, CPP

The American Institute of Architects
1735 New York Ave., NW
Washington, DC 20006
aia.org

The American Institute of Architects

Lugares de asistencia alternativos para atender casos de la enfermedad del coronavirus 2019 (COVID-19)

Instrumento para evaluar el estado de preparación V2.0

El presente instrumento para evaluar el estado de preparación presenta información resumida de situaciones no críticas (principios de diseño, pruebas de apoyo disponibles o traducción de las normas aplicables) en combinación con documentos del ámbito federal emitidos durante la crisis de la COVID-19. El contenido se preparó con la aportación conjunta de profesionales capacitados y experimentados de los ámbitos de la arquitectura, el diseño, la ingeniería, la ciencia, la seguridad de la vida, la salud y las operaciones de instalaciones hospitalarias.

Todos los estados y territorios de los Estados Unidos deberían estar preparados para la llegada de pacientes con COVID-19. Todos los hospitales y centros de salud pública deben asegurarse de que en los lugares de asistencia alternativos, tanto en un recinto de atención médica como en entornos comunitarios, se pueda gestionar la atención de los pacientes durante una respuesta de salud pública ante una pandemia con el fin de:

- potenciar al máximo la preservación de la vida;
- reducir el riesgo de propagación de agentes patógenos, incluido el SARS-CoV-2, dentro de la instalación;
- identificar rápidamente a los pacientes que requieren observación y aislamiento y trasladar al paciente o pacientes a la instalación correcta sobre la base de las determinaciones de la organización sanitaria o de la autoridad de salud pública con jurisdicción;
- atender a un mayor número de pacientes con condiciones diversas en el contexto de un brote/epidemia/pandemia que se extiende;
- garantizar la vigilancia y la gestión de la seguridad continua de las operaciones diarias (24 horas al día, 7 días a la semana);
- abordar la diversidad étnica y la adecuación cultural, así como las poblaciones marginadas, rurales, sin hogar y con trastornos mentales o de comportamiento.

La lista de verificación no describe requisitos obligatorios, pero sí destaca áreas importantes a evaluar por parte de hospitales y organismos de salud pública en la selección de lugares de asistencia alternativos donde prestar cuidados y tratamiento a los pacientes con COVID-19, o que tengan capacidad de sobrecarga. El objetivo es identificar la reutilización adecuada y de rápida adaptación de los entornos ya construidos, como centros de convenciones, estadios deportivos, centros comunitarios, hoteles, residencias estudiantiles u otros espacios para su ocupación durante la pandemia.

Descargo de responsabilidad: El presente documento se basa en la Lista de verificación integral para la preparación de los hospitales ante la enfermedad del coronavirus 2019 de los Centros para el Control y la Prevención de Enfermedades, adaptada para evaluar lugares de asistencia alternativos. Asimismo, el presente documento se preparó en un periodo de siete días de acción inmediata durante la “fase de alerta” de una respuesta a una pandemia (según la define el Programa Mundial de la OMS contra la Gripe respecto a la gestión de riesgos ante una pandemia de gripe). Este instrumento de evaluación es el resultado de la labor de un grupo de trabajo de respuesta a la COVID-19 del Instituto Americano de Arquitectos (*American Institute of Architects*) para educar e informar a arquitectos, profesionales de atención a la salud, profesionales de salud pública y autoridades con jurisdicción federal, estatal y/o local como parte de la respuesta a la pandemia emergente. Este material no pretende reemplazar las leyes, regulaciones o estándares profesionales aplicables existentes.

Selección del edificio: sí o no

Si el edificio carece de alguno de estos componentes básicos de construcción, es poco probable que la instalación actual sea la adecuada para brindar cuidados a los pacientes durante una pandemia ya que no se podrá modificar rápidamente.

Antes de finalizar la selección del lugar o de los lugares, se deberían examinar todas las consideraciones para determinar su idoneidad a la hora de sustentar la prestación de cuidados a los pacientes previstos.

	Sí	No
**Edad:** El lugar propuesto es un edificio relativamente nuevo, construido (o renovado) conforme a códigos contemporáneos (menos de 20 años de antigüedad).	○	○
**Seguridad de la vida:** Confirmar que el edificio cumple con los requisitos mínimos conforme al tipo de construcción y de evacuación según lo permitido por la autoridad con jurisdicción.	○	○
**Superficie adecuada:** Hay suficiente espacio para ajustarse a todas las necesidades de atención a los pacientes. (Evaluarlo según las consideraciones que se indican a continuación.)	○	○
**Suministro de agua municipal:** La calidad y la presión del agua sustentarán las operaciones clínicas seguras. (Evaluarlo según las consideraciones que se indican a continuación.)	○	○
**Enchufes eléctricos:** Hay un número suficiente de enchufes con toma de tierra para la seguridad de los pacientes y del personal. (Evaluarlo según las consideraciones que se indican a continuación.)	○	○

1.0 Condiciones generales para el uso del instrumento

1.1 Parámetros de referencia

Finalizado/convenido

1.1.1 Autoridad operante

- Confirmar qué autoridades tienen jurisdicción (por ejemplo, hospitales locales o sistemas sanitarios; sistemas hospitalarios con convenios de interoperabilidad; departamentos de salud/salud pública federales, estatales o locales; Departamento de Defensa de los Estados Unidos, la Agencia Federal para el Manejo de Emergencias (FEMA), la Guardia Nacional y milicias estatales; u otras combinaciones) ☐
- Confirmar si la autoridad puede cambiar durante el periodo de ocupación temporal ☐

1.1.2 Funciones administrativas sanitarias clave

- Establecer una estructura de mando para operaciones de atención de salud in situ con el fin de administrar y coordinar las cuestiones médicas y las labores de atención al paciente, y apoyar adecuadamente esas labores ☐
- Indicar y confirmar el apoyo a distancia necesario ☐

1.1.3 Gerencia según la instalación

- Establecer la estructura administrativa según la instalación para vigilar, mantener o administrar:

- las tecnologías de la información y las comunicaciones ☐
- la cadena de suministro ☐
- la posibilidad de peligro de incendio ☐
- el funcionamiento del equipo de climatización (HVAC) permanente y portátil ☐
- el suministro de gas médico (aire quirúrgico y oxígeno) ☐
- los protocolos para evitar infecciones ☐
- los residuos biopeligrosos ☐

• Elaborar procedimientos y administrar la evacuación y el traslado de pacientes y del personal durante situaciones de emergencia ☐

• Preparar mapas de salidas de emergencia y capacitar a los ocupantes en procedimientos de seguridad de la vida específicos para cada instalación ☐

1.1.4 Gestión de riesgos

• Determinar la autoridad del operador local para actuar en el mejor interés de los pacientes bajo su cuidado, evaluar el riesgo para los pacientes, priorizar los cuidados, asignar todos los recursos locales y ajustar el rumbo según sea necesario ☐

• Realizar y mantener actualizada una evaluación de riesgos de seguridad de la instalación (incluyendo el control de infecciones, la seguridad, la seguridad de los medicamentos, la prevención de infecciones, el manejo de pacientes y la posibilidad de lesiones asociadas con el comportamiento y la salud mental) ☐

• Cumplir con las leyes federales, estatales y locales (por ejemplo, la Ley para Estadounidenses con Discapacidades [ADA], la Ley de Portabilidad y Responsabilidad del Seguro de Salud [HIPPA], la Administración de Seguridad y Salud Ocupacional [OSHA]) al mismo tiempo que se garantiza la seguridad de la vida del paciente. (Las condiciones pueden diferir de las estrategias empleadas en la atención no pandémica.) ☐

• Confirmar con la autoridad con jurisdicción y el cuerpo de bomberos local si es necesario vigilar la posibilidad de incendios las 24 horas del día, 7 días a la semana, debido a las deficiencias en la seguridad de los edificios y de las alarmas contra incendios. ☐

1.1.5 Apoyo profesional para la selección y establecimiento de lugares de asistencia alternativos

• Implicar a arquitectos e ingenieros con licencias, formación y experiencia en el ámbito de la salud, así como a profesionales del diseño familiarizados con el tipo de edificio de asistencia alternativo que se esté considerando ☐

1.2 Recursos temporales

- Confirmar si también se le exigirá al operador que provea personal médico temporal, un generador eléctrico, equipo de climatización (HVAC) adicional, producción de gas modular, instalaciones con duchas, inodoros y lavabos, alojamiento para el personal, alimentos y suministros ☐

1.3 Recomendaciones en curso

- Estas recomendaciones respaldan una solución a favor de una instalación de respuesta rápida, y cambian según lo justifiquen las condiciones y los antecedentes. ☐

2.0 Requisitos funcionales

2.1 Concepto para el funcionamiento

Sí/Convenido

- Se identifica el nivel de cuidado de los pacientes como:
  - ambulatorio (capaz de cuidarse a sí mismo), ☐
  - grave (incapaz de cuidarse a sí mismo), ☐
  - crítico (por ejemplo, que necesita mantenimiento de las funciones vitales/ventilación mecánica). ☐
- Se ha identificado el diagnóstico del paciente para su tratamiento:
  - COVID-19 positivo (COVID), ☐
  - COVID-19 negativo (no COVID), ☐
  - casos transitorios (por ejemplo, asintomáticos, personas bajo observación, posteriores al alta). ☐
- Elaborar un plan para determinar dónde tendrán lugar tanto el diagnóstico como el tratamiento ☐
- Elaborar un plan para proporcionar cuidados a pacientes con trastornos mentales y de comportamiento no esperados (por ejemplo, observación, áreas de diseño seguras, traslados) ☐

2.2 Atención al paciente in situ

2.2.1 Selección del lugar/ubicación (ver también 2.2.6, 2.3.1)

- El lugar está situado muy cerca del personal de respuesta inmediata. ☐
- El lugar está ubicado cerca de un hospital. ☐
- Confirmar la disponibilidad de estacionamiento para el personal, las ambulancias, los servicios de entrega y los de recogida de deshechos ☐

2.2.2 Aislar los procedimientos de COVID-19

- Los procedimientos relacionados con la COVID pueden estar separados de otras actividades en la instalación. ☐
- Establecer un flujo unidireccional de pacientes, personal y materiales de limpio a contaminado ☐
- Aislar los espacios de apoyo para el personal de los espacios de atención al paciente ☐
- Establecer un plan para realizar cambios al tipo de atención que el paciente recibe durante el funcionamiento del lugar de asistencia alternativo ☐

2.2.3 Se adecúa el espacio para pacientes para:

- un número proyectado de ______ ocupantes, con:
  - observación directa o a distancia de los pacientes, ☐
  - privacidad de los pacientes, ☐
  - la entrada de luz diurna en el espacio para pacientes (preferiblemente hay ventanas en las habitaciones de los pacientes), ☐
  - uso del inodoro y las duchas por parte del paciente, ☐
  - lavamanos para el personal, ☐
  - espacio/conexiones para los respiradores, postes de infusión intravenosa, monitores, eliminación de riesgos biológicos, etc. ☐
- Espacio para el carrito de almacenamiento y el contenedor de desechos (reciclaje) del equipo de protección personal disponible inmediatamente. ☐
- Espacio para que el personal pueda ponerse y quitarse el equipo de protección personal en las áreas de atención al paciente con espacio para que el camarada de apoyo lo supervise (o a través de las observación por cámara). ☐
- Zona segura para las pertenencias de los pacientes ☐

2.2.4 Las áreas de trabajo de los equipos del personal de enfermería y médico necesitan disponer de:

- un espacio de documentación para médicos y personal de enfermería, ☐
- un espacio para la comunicación de los equipos de enfermería y médico, ☐
- un carrito de reanimación cardiopulmonar, ☐
- un lavamanos para el personal, ☐

- un espacio para pruebas en el área de cuidados (por ejemplo, glucómetros, i-STAT, equipo de gasometría arterial), ☐
- un anunciador de llamadas del personal de enfermería, ☐
- monitores de vigilancia. ☐

2.2.5 El apoyo al personal de enfermería necesita incluir:

- una sala de preparación de medicamentos (segura) o una unidad autónoma de dispensación con iluminación adecuada, ☐
- área(s) de alimentación con fregadero, mostrador de preparación, refrigerador, máquina de hielo y estación de lavado de manos, ☐
- sala(s) de materiales limpios, ☐
- una sala donde guardar materiales sucios equipada con un fregadero y un mostrador de trabajo que se pueda limpiar, ☐
- un cuarto para almacenar ropa limpia, ☐
- un cuarto para almacenar equipo y materiales, ☐
- un cuarto para servicios ambientales, ☐
- almacenamiento seguro para las pertenencias del personal, ☐
- inodoros, lavabos y duchas para el personal, ☐
- una zona de almacenamiento de equipo de protección personal en toda la instalación o unidad (protegida según sea necesario), ☐
- descontaminación y reparación de respiradores, ☐
- una sala de descanso o zona de reposo con acceso a luz diurna (cuando sea posible), ☐
- dormitorios para personal de guardia. ☐

2.2.6 Admisión y alta

- Establecer el espacio necesario para los protocolos de seguridad ☐
- Proporcionar un lugar cubierto para las ambulancias cuando traen a pacientes ☐
- Adaptación para transportar a un paciente relacionado con un caso de enfermedad o muerte ☐

2.2.7 Admisión y alta si se utiliza para pacientes sin cita previa

- Registro separado para pacientes sin cita previa o de paso en automóvil ☐
- Área de evaluación cerrada, cubierta o de paso en automóvil (por ejemplo, para la toma de muestras y la evaluación) ☐
- Salida separada para el paciente, alejada de la entrada ☐

2.2.8 Final de la vida

- Dar acceso virtual a la familia (wi-fi adecuado) ☐
- Proporcionar un área para visitas al final de la vida (conforme a las políticas y protocolos permitidos) ☐
- Proporcionar una ventana con vista al exterior, si hay una disponible (si el paciente está consciente) ☐

2.3 Apoyo general

2.3.1 El apoyo in situ necesita incluir:

- seguridad del lugar, ☐
- entrada y salida separadas designadas para el personal, coherentes con la evaluación de riesgos de seguridad, ☐
- recepción y logística, ☐
- farmacia satélite y seguridad conexa, ☐
- un laboratorio STAT (véase también el espacio para pruebas en el área de cuidados en el apartado 2.2.4 Apoyo al personal de enfermería), ☐
- depósito de cadáveres (móvil, si fuera necesario), ☐
- gestión de desechos normales y de riesgo biológico, ☐
- sala limpia para la descontaminación de respiradores, ☐
- entrega y distribución de alimentos. ☐

2.3.2 Apoyo fuera de la instalación

- Si no se proporciona in situ, confirmar los acuerdos de gestión entre organizaciones para:
  - el procesamiento estéril, ☐
  - el procesamiento de ropa blanca, ☐
  - el almacenamiento de equipo y material general y de logística, laboratorio general, ☐
  - la farmacia general, ☐
  - el laboratorio, ☐
  - el servicio de alimentación. ☐

2.3.3 Gerencia

- Áreas de apoyo a la gerencia de la instalación ☐

2.3.4 Área de apoyo al cuidador para descansar, aislarse o cuarentenarse

- Confirmar el lugar o los lugares de alojamiento in situ temporal (para personal médico, de enfermería y de apoyo) ☐
- Evaluar la posibilidad de contratar a hoteles del área o residencias estudiantiles cercanos ☐

3.0 Modificaciones de las instalaciones necesarias en virtud del diseño y la construcción

3.1 Arquitectónicas

3.1.1 Tipo de construcción

- Evitar la construcción combustible ☐
- Se prefiere: NFPA 101, Código de seguridad de la vida tipo II o mayor nivel de protección contra incendios y de seguridad de los ocupantes ☐

3.1.2 Seguridad pasiva

- Confirmar si las escaleras permiten evacuar a los pacientes en una camilla de rescate, si los pacientes son incapaces de cuidarse ellos mismos ☐
- Confirmar las vías de salida para la clasificación apropiada en materia de incendio y humo de los recintos ☐
- Estudiar la compartimentación respecto de incendios y humo en función de la densidad de pacientes (especialmente si no hay o hay pocas salidas para el humo) ☐
- Confirmar que los cuartos de almacenamiento general de >100 pies cuadrados y los cuartos de almacenamiento de oxígeno están clasificados para los incendios, incluyendo el cierre positivo ☐
- Confirmar que como mínimo hay una salida horizontal ☐
- Proporcionar como mínimo dos salidas por cada piso donde hay pacientes ☐
- Señales de salida e iluminación de salida adecuadas ☐

3.1.3 Protegerse in situ

- Cuando los pacientes no sean capaces de cuidarse de ellos mismos, evaluar los planes de seguridad de vida para protegerse in situ o elaborar un plan activo de seguridad vital basado en la operación del lugar ☐

3.1.4 Geometría del edificio

- Confirmar la configuración (paredes, puertas, pasillos, etc.) para que el personal pueda mover fácilmente las camillas y el equipo médico ☐

- Confirmar la visibilidad para que el personal mantenga las líneas de visión en las áreas de cuidado de los pacientes con el fin de vigilar activamente a los pacientes, así como para reducir el riesgo de incidentes de comportamiento (por ejemplo, fuga, agresión, en caso de comorbilidad de comportamiento o trastorno mental). ☐

3.1.5 Partes del edificio

- Disponer de entradas o puertas para las habitaciones de pacientes que:
  - tengan ventanillas para observar desde fuera de la habitación, ☐
  - contengan esporas en aerosol, ☐
  - tengan suficiente espacio para la entrada y salida de equipos y camillas. ☐
- Proporcionar acabados lavables y fáciles de limpiar en las áreas de los pacientes (evaluar quitar los acabados existentes, como revestimientos de paredes, cortinas y alfombras) ☐
- Proporcionar suelos de superficie dura y antideslizante ☐

3.1.6 Equipo y mobiliario

- Utilizar camas y sillas para pacientes que puedan ser desinfectadas ☐
- Evaluar el espacio para el mobiliario y el equipo médico necesarios ☐
- Sacar el equipo o el mobiliario que no se utilice directamente para el cuidado de los pacientes ☐

3.1.7 Circulación

- Los ascensores deberían permitir al personal trasladar a un paciente en una camilla o, si es ambulatorio, en una silla de ruedas. ☐
- Considerar la posibilidad de separar el tráfico de personal y de pacientes en los ascensores ☐

3.2 Estructurales

- Confirmar que el sistema estructural existente puede soportar la carga estructural (activa) adicional a consecuencia del aumento en el número de ocupantes, equipo médico y cualquier equipo adicional ubicado en la azotea ☐

3.3 Mecánicas

3.3.1 Aislamiento y presión negativa

- Si es necesario por cuestiones clínicas, hacer preparativos para disponer de una sala, zona o piso de presión negativa ☐

- Si es posible y necesario por cuestiones clínicas, utilizar o crear salas de aislamiento para infecciones de transmisión aérea ☐
- Cuando sea posible, ubicar el extractor del sistema de ventilación cerca de la cabeza del paciente ☐
- Asegurar que los espacios contiguos están protegidos contra la contaminación cruzada ☐

3.3.2 Cambios de aire y filtración

- Cuando sea posible, la preferencia es hacer 10 cambios de aire por hora, más 2 cambios de aire exterior. ☐
- Cuando sea posible, la preferencia es la de utilizar filtros MERV 7 en las zonas donde hay pacientes y filtros finales MERV 14. ☐

3.3.3 Probar y ajustar

- Realizar pruebas de posconstrucción o renovación y ajuste del sistema de climatización ☐

3.3.4 Posibles limitaciones del sistema existente

- Considerar las unidades de pared y el extractor del baño respecto a su capacidad de extracción, filtración y tasas de cambio de aire existentes (Estas pueden verse afectadas por el tamaño de los conductos.) ☐
- Considerar unidades para azoteas montadas con calefacción eléctrica para proporcionar ventilación adicional ☐
- Considerar la posibilidad de mejorar los sistemas de ventilación de los edificios (por ejemplo, ventiladores de mayor volumen) para mantener la presión negativa ☐
- Evaluar los controles de humedad para proporcionar niveles de humedad óptimos para el cuidado y tratamiento eficaces de los pacientes ☐
- Considerar la posibilidad de utilizar unidades de reciclaje del aire con filtración HEPA en las habitaciones de los pacientes ☐

3.3.5 Control del humo

- Confirmar las capacidades del sistema de control de humo. Si no es posible, la cantidad de pacientes puede ser limitada. ☐

3.4 Eléctricas

3.4.1 Electricidad

- Proporcionar circuitos eléctricos según las exigencias de los equipos ubicados donde estén los pacientes y el personal ☐
- Confirmar la ubicación de las cajas eléctricas en los pisos para la distribución general de electricidad ☐

3.4.2 Disponibilidad de electricidad derivada

- Confirmar que los circuitos derivados están disponibles para respaldar las operaciones de atención al paciente ☐

3.4.3 Electricidad de emergencia

- En función de las necesidades de los pacientes (es decir, ambulatorios o de cuidados intensivos), proporcionar circuitos de electricidad ininterrumpidos para cuidados intensivos y de seguridad de la vida para sustentar las zonas de atención al paciente durante al menos 24 horas para los servicios esenciales, entre ellos:
  - equipo médico de soporte vital y equipo y frigoríficos para el almacenamiento de medicamentos, ☐
  - enchufes para el equipo médico en las áreas de atención al paciente y en las cabeceras, ☐
  - sistema de llamada al personal de enfermería, ☐
  - tecnología de la información (relativa a los pacientes, comunicación del equipo médico e infraestructura), ☐
  - iluminación en las salidas, ☐
  - sistema de alarma de incendios, ascensores, infraestructura de la tecnología de la información, ☐
  - ascensores, ☐
  - interruptor de transferencia automática. ☐

3.4.4 Iluminación

- Proporcionar iluminación (lámparas herméticas) y lámparas portátiles de examen para apoyar la operación clínica ☐
- Verificar la iluminación de emergencia para las salidas ☐

3.5 Fontanería/gas médico

3.5.1 Gas médico

- Proporcionar adecuaciones para el oxígeno, el aire médico y la aspiración para sustentar la intubación y el funcionamiento de los respiradores para dar cabida a la alta demanda o total ☐
- Confirmar la disponibilidad de espacio para tanques generadores de oxígeno modulares temporales, cuando sean necesarios ☐
- Confirmar la disponibilidad de un contenedor de almacenamiento debidamente clasificado contra incendios o de una sala para el oxígeno embotellado ☐
- Confirmar la disponibilidad de almacenamiento para tanques de oxígeno vacíos ☐

3.5.2 Agua

- Proporcionar agua potable para respaldar la operación clínica. (Podría incluir la desinfección de la red de agua y del sistema de distribución de agua además de pruebas analíticas del edificio para confirmar que el sistema de gestión del agua es seguro para las operaciones de cuidados a los pacientes.) ☐
- Si se exigiera, elaborar un plan para procesar el agua para la descontaminación o esterilización in situ ☐
- Elaborar un plan para la gestión permanente del sistema de agua que incluya las descargas de inodoros, el control de la temperatura y los niveles de desinfectante residuales ☐

3.6 Protección contra incendios/seguridad de la vida

- Confirmar las condiciones y expectativas de seguridad en caso de incendio y de la vida con las autoridades con jurisdicción locales ☐
- Confirmar la presencia de un sistema de alarma contra incendios y su funcionamiento para apoyar la atención a los pacientes. Si no hay sistema de alarma contra incendios, reevaluar la idoneidad del edificio para la respuesta ante una pandemia, o instalar un sistema de alarma contra incendios ☐
- Preferencia: Sistema de extinción de incendios totalmente automático ☐
- Confirmar el suministro y el espaciamiento adecuados de los extintores de incendios ☐

3.7 Comunicación

3.7.1 Tecnología de la información

- Confirmar la existencia de una infraestructura de la información segura que sea adecuada o instalarla para apoyar la atención a los pacientes, que incluya el cumplimiento de la HIPAA ☐

3.7.2 Comunicación de los servicios médicos de emergencia

- Confirmar la existencia de un sistema de comunicación para servicios médicos de emergencia adecuado o instalarlo para apoyar la atención a los pacientes que cumpla con la seguridad de la HIPAA ☐

3.7.3 Llamada al personal de enfermería o comunicación

- Confirmar la existencia de un sistema seguro adecuado para llamar al personal de enfermería o instalarlo para apoyar la atención a los pacientes (o un sistema similar de comunicación del paciente al personal de enfermería) ☐
- Establecer un método para la comunicación del equipo médico (por ejemplo, móvil o inalámbrico) ☐

3.7.4 Alarma contra incendios

- Confirmar la existencia de un sistema de alarma contra incendios conectado a los equipos de emergencia locales o instalarlo ☐
- Comprobar la disponibilidad de unidades de alarmas contra incendios con pulsadores palanca ☐

3.7.5 Sistemas de seguridad

- Evaluar las necesidades en materia de seguridad y proporcionar un sistema de seguridad según corresponda (cámaras, monitores, detectores de movimiento, alarmas para puertas y ventanas, etc.) ☐
- Considerar sistemas inalámbricos donde se necesiten ☐

4.0 Observaciones adicionales

4.1 Grupos étnicamente diversos o marginados

- Examinar las necesidades de espacio para abordar la adecuación cultural ☐
- Considerar la posibilidad de acomodar las prácticas espirituales y culturales específicas de la comunidad ☐
- Considerar la accesibilidad de los lugares de asistencia alternativos y las opciones de transporte, y dentro o adyacentes a los complejos de viviendas públicas ☐
- Proporcionar espacios dedicados a los trabajadores sociales y a los servicios de traducción de idiomas ☐

4.2 Poblaciones de zonas rurales

- Considerar la accesibilidad y transporte a lugares de asistencia alternativos, evaluando los tiempos de conducción y las distancias ☐
- Considerar la necesidad de transporte en helicóptero ☐
- Considerar la necesidad de vivienda y alojamiento para el personal de salud y las familias de los pacientes ☐
- Evaluar la necesidad de servicios de ambulancia para transportar a pacientes de mayor gravedad ☐
- Comprobar los sistemas de comunicación (teléfonos móviles y repetidores para mejorar la comunicación inalámbrica) ☐
- Comprobar que las necesidades de infraestructura crítica (alcantarillado, tanques sépticos, agua potable y electricidad) sean adecuadas para la demanda prevista ☐

4.3 Poblaciones sin hogar y sin refugio

- Proporcionar lugares con baños y duchas ubicados cerca de la entrada ☐
- Designar sitios donde guardar las pertenencias, bicicletas, etc. ☐

4.4 Pacientes con trastornos mentales y de comportamiento

- De conformidad con la sección 1.1.4, realizar una evaluación de riesgos de seguridad de todos los lugares accesibles a posibles pacientes con trastornos mentales y de comportamiento a fin de evitar que se autolesionen y que lesionen a otros ☐
- Si se identifican pacientes con trastornos mentales y de comportamiento, proporcionar espacio para observación individual continua hasta su trasladado o alta ☐
- Considerar la posibilidad de tener salas seguras, si es oportuno en función del plan operacional identificado ☐

Pautas adicionales

Para obtener material de referencia adicional, véase https://bit.ly/C19HealthcareArch

Contribuciones del grupo de trabajo y de los autores

Miembros del grupo de trabajo

(MS) Molly M. Scanlon, PhD, FAIA, FACHA – presidenta
(DA) Diana Anderson, MD, ACHA
(EE) Eve A. Edelstein, PhD, MArch, Assoc. AIA, EDAC, FAAA
(JF) John Fowler, AIA, NCARB, EDAC, LEED AP
(WH) William Hercules, FAIA, FACHA, FACHE
(EP) Erin Peavey, AIA, EDAC, LEED AP
(YS) Yiselle Santos, AIA, LSSYB, WELL AP, LEED AP
(ET) Ellen Taylor, PhD, AIA, MBA, EDAC
(KW) Kirsten Waltz, AIA, ACHA, EDAC, LEED AP
(MW) Marvina Williams, RN, BSN, LSSBB
(FZ) Frank Zilm, DArch, FAIA, Emérito FACHA
(DP) Doug Paul, personal del AIA

Instrumento para evaluar la preparación de los lugares de asistencia alternativos para atender casos de COVID-19
Las siguientes son las contribuciones sustanciales de cada uno de los autores: conceptualización, MS; análisis de códigos formales y de estándares, JF, WH, KW y ET; administración de proyectos, ET, MS y DP.

Reuniones
El grupo de trabajo celebró reuniones diarias del 24 de marzo al 2 de abril de 2020 para preparar el Informe sobre el impacto en la salud N.° 1 y productos de trabajo conexos para su examen, consenso y aprobación. Durante el mismo periodo se celebraron reuniones adicionales de grupos más pequeños para cada producto de trabajo. Se prepararon actualizaciones hasta el 22 de abril de 2020 para la publicación de una versión actualizada.

Contribuciones adicionales al Instrumento para evaluar la preparación de los lugares de asistencia alternativos para atender casos de COVID-19
Amanda Abney, Associate AIA, EDAC
Patricia Acevedo, AIA
Javan Arroyo
Joe Bonanno
Gabrielle Bullock, FAIA, NOMAC, IIDA, LEED AP
Daniel Caron, PE, LEED AP
Jason Costello, AIA, EDAC
Ashleigh Cotter, CID, CHID, IIDA, LEED AP BD+C, CLGB
Rachael Farrell, EDAC, LSSGB, LEED AP, BD+C
David Frum, FAIA, FACHA
Ruth Fanning, MD
Angel Gomez
Jeff Hankin, PE
Anita Honkanen, MD
Nathan Howell, AIA
Jennifer Burke Jackson, AIA, LEED AP
Isaac Karley
Ed Ledoux
Christopher Lynch, PE
Zach Mathern
Don MacAlister, CHPA
Todd Medd, AIA, NCARB
Christopher O'Brien, AIA, ACHA, EDAC, LEED AP
Sam Olbekson, AIA, NCARB, AICAE
John P. Riordan, MD
Nick Schumacher, PE
Michael Sheerin, PE, LEED AP
Hannah Shultz
Tom Smith, CHPA, CPP
Rick Taylor, RCDD
Kevin Tuohey, CHPA
Scott Waltz, RA
Julie Wellik
Tony York, CHPA, CPP

The American Institute of Architects

Ferramenta de avaliação do estado de preparação V2.0

Esta ferramenta de avaliação do estado de preparação contém informações compiladas em situações sem crise (princípios de *design*, evidências comprobatórias disponíveis, ou traduções dos padrões aplicáveis) em conjunto com documentos emitidos durante a crise da Covid-19. O conteúdo foi preparado com informações abalizadas de profissionais treinados e experientes da área de saúde: arquitetos, projetistas, engenheiros, cientistas, consultores em segurança vital, médicos e enfermeiros e operadores de instalações hospitalares.

Todos os estados e territórios dos EUA devem estar preparados para a chegada de pacientes com a Covid-19. Todos os hospitais e órgãos de saúde pública precisam garantir que locais alternativos de atendimento, tanto um campus de atendimento de saúde quanto instalações comunitárias, estejam preparados para receber pacientes durante uma resposta de saúde pública à pandemia a fim de:

- maximizar a preservação da vida;
- mitigar os riscos de propagação de patógenos, inclusive a SARS-CoV-2, dentro das instalações;
- identificar prontamente os pacientes que requeiram observação e isolamento, enviando-os a instalações adequadas segundo determinação da organização de saúde ou autoridade de saúde pública com jurisdição;
- cuidar de um número maior de pacientes com condições diferenciadas no contexto de uma escalada de surto/epidemia/pandemia; e
- garantir monitoramento e gerenciamento constantes da segurança para funcionamento ininterrupto;
- levar em consideração a diversidade étnica e competência cultural, bem como as populações marginalizadas, rurais, em situação de rua e com problemas mentais/comportamentais.

A lista de verificação não descreve os requisitos obrigatórios, mas destaca as áreas importantes para avaliação dos hospitais e órgãos de saúde pública na seleção de locais alternativos de atendimento para o cuidado e tratamento de pacientes com Covid-19 ou que tenham ultrapassado a capacidade. O objetivo é identificar estruturas existentes para reutilização adequada com adaptação rápida, como centros de convenção, estádios esportivos, centros comunitários, hotéis, dormitórios e outros espaços que possam ser ocupados durante a pandemia.

Aviso: este documento segue o modelo da Lista de Verificação Detalhada de Preparação Hospitalar para a Doença do Coronavírus 2019 dos Centros de Controle e Prevenção de Doenças, com adaptação para a avaliação de locais alternativos de atendimento. Além disso, este documento foi preparado em um período de sete dias de resposta rápida durante a “fase de alerta” em reação a uma pandemia (conforme a definição do Programa Global de Gestão de Risco de Influenza Pandêmica da OMS). A ferramenta de avaliação é o resultado do grupo de trabalho em resposta à Covid-19 do Instituto Americano de Arquitetos (*American Institute of Architects-AIA*) para educar e informar arquitetos, profissionais de atenção de saúde, profissionais de saúde pública e autoridades com jurisdição federal, estadual e/ou municipal competente em resposta à pandemia emergente. Este material não tem por objetivo substituir os regulamentos, as leis ou os padrões profissionais existentes e válidos.

Escolha de estruturas adequadas/não adequadas

Se a edificação não contar com estes componentes básicos de construção, dificilmente será adequada para o atendimento de pacientes durante uma pandemia, já que não pode ser rapidamente alterada.

Antes de finalizar a escolha do local, todas as considerações precisam ser analisadas quanto à sua adequação para atender à população de pacientes a que se destina.

	Sim	Não
**Idade.** O local proposto é um edifício mais novo, erguido (ou reformado) segundo códigos contemporâneos (menos de vinte anos).	○	○
**Segurança vital.** Confirmar se a edificação atende aos requisitos mínimos de construção e saídas, segundo a autoridade com jurisdição na área.	○	○
**Área útil adequada.** Há espaço suficiente para acomodar todas as funções necessárias ao atendimento de pacientes (avaliar segundo as considerações a seguir).	○	○
**Abastecimento municipal de água.** A qualidade e pressão da água darão suporte à segurança das operações clínicas (avaliar segundo as considerações a seguir).	○	○
**Tomadas elétricas.** Há um número suficiente de tomadas aterradas para a segurança de pacientes e funcionários (avaliar segundo as considerações a seguir).	○	○

1.0 Condições gerais para o uso da ferramenta

1.1 Parâmetros de linha de base	Concluído/ Acordado
**1.1.1 Autoridade operacional**	
• Confirmar quais as autoridades que têm jurisdição (p. ex., hospitais/sistemas de saúde locais; sistemas hospitalares com acordos operacionais entre eles; departamento de saúde/saúde pública federal, estadual ou municipal; Ministério da Defesa, Agência Federal de Gerenciamento de Emergências [FEMA, na sigla em inglês], Guarda Nacional, polícia estadual; outras combinações)	☐
• Confirmar se a autoridade pode mudar durante o curso da ocupação temporária	☐
**1.1.2 Principais papéis administrativos de saúde**	
• Estabelecer uma estrutura de liderança nas operações de saúde no local para dirigir e coordenar as questões médicas e de atendimento ao paciente, e dar apoio adequado a essas operações	☐
• Identificar e confirmar o apoio remoto necessário	☐
**1.1.3 Administração específica das instalações**	
• Estabelecer uma estrutura administrativa baseada nas instalações para monitorar, manter ou gerenciar:	
– TI e comunicações	☐
– a cadeia de abastecimento	☐
– o potencial de riscos de incêndio	☐

- a operação de equipamentos AVAC permanentes ou portáteis ☐
- o abastecimento de gases medicinais (ar e oxigênio cirúrgico) ☐
- protocolos de prevenção de infecções ☐
- resíduos de risco biológico ☐

- Desenvolver procedimentos e administrar evacuação/movimento de pacientes e funcionários em condições de emergência ☐
- Criar mapas para saída de emergência e treinar os ocupantes em procedimentos de segurança vital específicos para as instalações ☐

1.1.4 Gestão de risco

- Estabelecer a autoridade do operador local para agir no melhor interesse dos pacientes sob seus cuidados, avaliar o risco dos pacientes, priorizar tratamentos, alocar/designar todos os recursos locais e ajustar o curso conforme for necessário ☐
- Realizar e manter atualizada uma avaliação de riscos de segurança (incluindo controle de infecção, segurança, segurança dos medicamentos, prevenção de infecção, manejo de pacientes e a possibilidade de ferimentos associados à saúde comportamental/mental) ☐
- Obedecer as leis federais, estaduais e municipais (p. ex., Lei dos Americanos Portadores de Deficiências–ADA, Lei de Portabilidade e Responsabilidade do Seguro de Saúde–HIPAA, Administração de Segurança e Saúde Ocupacional–OSHA), garantindo ao mesmo tempo a segurança vital dos pacientes (as condições podem ser diferentes das estratégias empregadas nos casos de cuidados não pandêmicos) ☐
- Confirmar com a autoridade jurisdicional e com o corpo de bombeiros local se é necessário haver monitoramento de incêndio, e se ele está disponível, 24 horas ao dia devido a deficiências existentes no edifício quanto a segurança vital/alarme contra incêndio ☐

1.1.5 Apoio profissional para a escolha e desenvolvimento de locais alternativos de atendimento

- Trabalhar com arquitetos e engenheiros certificados, treinados e experientes na área de saúde e com projetistas profissionais que conheçam o tipo de edificação alternativa de atendimento em consideração ☐

1.2 Recursos temporários

- Confirmar se o operador também terá que fornecer equipe médica temporária, gerador elétrico, equipamento AVAC adicional, geração modular de gás, toaletes e chuveiros, acomodação para o pessoal, alimentação e materiais ☐

1.3 Evolução das recomendações

- Essas recomendações apoiam uma solução de resposta rápida para instalações, e evoluem conforme as condições e os precedentes ☐

2.0 Requisitos funcionais

2.1 Conceito para o funcionamento

Sim/Acordado

- Nível de cuidado do paciente é identificado como:
  - ambulatorial (capaz de autopreservação) ☐
  - agudo (incapaz de autopreservação) ☐
  - crítico (p. ex., necessita de suporte vital/ventilação mecânica) ☐
- O diagnóstico de tratamento do paciente foi identificado como:
  - Covid-19 positivo (Covid) ☐
  - Covid-19 negativo (não-Covid) ☐
  - casos em transição (p. ex., assintomáticos, pessoas sob observação, pós-alta) ☐
- Desenvolver um plano para diagnóstico e tratamento ☐
- Desenvolver um plano para tratamento de pacientes com problemas mentais e comportamentais imprevistos (p. ex., observação, áreas seguras, transferência) ☐

2.2 Atendimento a pacientes no local

2.2.1 Escolha do local/localização (ver também 2.2.6, 2.3.1)

- O local está próximo de socorristas ☐
- O local é de fácil acesso a um hospital ☐
- Confirmar estacionamento para funcionários, ambulâncias, entregas e expurgo de resíduos ☐

2.2.2 Isolar as operações ligadas à Covid-19

- Operações ligadas à Covid-19 podem ser separadas de outras atividades nas instalações ☐
- Estabelecer um fluxo de mão única para pacientes, funcionários e materiais, que passe da área não contaminada para a contaminada ☐
- Isolar espaços de apoio aos funcionários de espaços de atendimento a pacientes ☐
- Estabelecer um plano para a transição do tipo de atendimento ao paciente durante o funcionamento do local alternativo de atendimento ☐

2.2.3 Os espaços dedicados a pacientes são dimensionados para acomodar

- Um número previsto de ______ ocupantes com:
  - monitoramento direto ou remoto do paciente ☐
  - privacidade do paciente ☐
  - entrada de luz do dia no espaço dedicado ao paciente (de preferência, janelas no quarto do paciente) ☐
  - toalete e banheiro para o paciente ☐
  - pia para lavagem das mãos do pessoal ☐
  - espaço/conexões para ventiladores, haste de suporte de IV, monitores, expurgo de material biológico de risco, etc. ☐
- Espaço para o carrinho de armazenamento e receptáculo (para reciclagem) de equipamento de proteção individual (EPI) disponível no momento ☐
- Lugar para para o pessoal pôr e remover as peças de EPI em áreas de atendimento a pacientes com espaço para um sistema de supervisão dos colegas (ou mediante observação por câmera) ☐
- Área segura para os pertences do paciente ☐

2.2.4 Os espaços para os postos de enfermagem/médicos precisam acomodar:

- área para documentação médica e de enfermagem ☐
- área para comunicação da equipe de enfermagem/clínica ☐
- carrinho de ressuscitação cardiopulmonar ☐
- pia para a lavagem das mãos do pessoal ☐
- um espaço para testes no local de atendimento (p. ex., glicosímetro, i-STAT, equipamento de gasometria arterial) ☐
- anunciador de chamada do pessoal de enfermagem ☐
- monitores de vigilância ☐

2.2.5 O suporte ao pessoal de enfermagem deve incluir:

- disponibilização de uma sala de preparação de medicamentos (segura) ou um dispensário isolado com iluminação adequada ☐
- copa com pia, bancada para preparação, geladeira, máquina de gelo e pia para lavar as mãos ☐
- cômodo(s) para utensílios de limpeza ☐
- sala para guardar materiais sujos com uma pia lavável e uma bancada que possa ser limpa ☐

- sala para armazenamento de toalhas e lençóis limpos ☐
- sala para armazenamento de equipamentos e materiais ☐
- sala de serviços ambientais ☐
- local para guarda segura de pertences do pessoal ☐
- instalações sanitárias e chuveiros para os funcionários ☐
- área para o armazenamento de EPI da instalação/unidade (segura, se necessário) ☐
- descontaminação e conserto de ventiladores ☐
- sala de recreação ou descanso com acesso à luz do dia (quando possível) ☐
- quarto para o descanso de plantonistas ☐

2.2.6 Admissão e alta

- Estabelecer o espaço necessário para protocolos de segurança ☐
- Disponibilizar uma área coberta para a chegada de ambulâncias ☐
- Acomodação para transferência de pacientes no caso de doença ou óbito ☐

2.2.7 Admissão e alta de pacientes ambulatórios

- Área separada para o registro de paciente ambulatório ou no carro (*drive through*) ☐
- Área de triagem fechada, coberta ou de passagem para carros (p. ex., para coleta de amostra e avaliação) ☐
- Saída separada de pacientes, longe da entrada de pacientes ☐

2.2.8 Final da vida

- Acomodar o acesso virtual da família (wi-fi adequado) ☐
- Oferecer áreas de visita para o final da vida (com base em políticas e protocolos permitidos) ☐
- Disponibilizar uma janela externa se possível (e se o paciente estiver consciente) ☐

2.3 Suporte geral

2.3.1 O suporte no local deve incluir:

- segurança do local ☐
- área separada para entrada e saída de funcionários de acordo com a avaliação de riscos de segurança ☐
- recepção e logística ☐

- farmácia satélite e segurança pertinente ☐
- laboratório STAT (ver também local em 2.2.4 para suporte à enfermagem) ☐
- necrotério (móvel, se necessário) ☐
- manejo de resíduos normais e de risco biológico ☐
- oficina para descontaminação e limpeza de ventiladores ☐
- entrega e preparação de alimentos ☐

2.3.2 Suporte externo

- Caso não esteja disponível no local, confirmar os acordos operacionais entre organizações para:
  - processamento de esterilização ☐
  - processamento de toalhas e lençóis ☐
  - armazenamento e logística para equipamentos e materiais gerais, laboratório geral ☐
  - farmácia geral ☐
  - laboratório ☐
  - serviços de alimentação ☐

2.3.3 Administração

- Áreas de apoio à gerência do local ☐

2.3.4 Área de apoio aos cuidadores para descanso, condições de auto-isolamento/quarentena

- Confirmar o(s) local(is) para alojamento temporário (pessoal médico, de enfermagem e de apoio) ☐
- Analisar a possibilidade de contratação de hotéis e outros que ofereçam alojamento nas adjacências ☐

3.0 Modificações requeridas nas instalações no âmbito de projeto/construção

3.1 Arquitetônicas

3.1.1 Tipo de construção

- Evitar construções combustíveis ☐
- Preferíveis: NFPA 101, nível de proteção contra incêndio e segurança vital dos ocupantes Life Safety Code tipo II ou maior ☐

3.1.2 Segurança vital passiva

- Confirmar se as escadas permitem a evacuação em macas, se os pacientes forem incapazes de autopreservação ☐
- Confirmar as vias de saída para classificação adequada da edificação quanto a incêndio e fumaça ☐
- Rever a compartimentação de incêndio e fumaça com base no número de pacientes (principalmente se não houver amortecedores de fumaça ou se estes forem limitados) ☐
- Confirmar se as salas de armazenamento geral têm mais de 10 m^2 e se as salas de armazenamento de oxigênio têm classificação contra incêndio, incluindo fechos positivos ☐
- Confirmar que haja pelo menos uma saída horizontal ☐
- Proporcionar ao menos duas saídas por andar onde houver pacientes ☐
- Adequar a sinalização e iluminação das saídas ☐

3.1.3 Defesa no local

- Quando os pacientes forem incapazes de autopreservação, avaliar planos de segurança vital para defesa no local ou desenvolver um plano ativo com base operacional para segurança vital ☐

3.1.4 Geometria da edificação

- Confirmar a disposição (paredes, portas, corredores, etc.) para permitir que o pessoal movimente facilmente macas e equipamentos médicos ☐
- Confirmar visibilidade para que o pessoal mantenha linhas de visão em áreas de atendimento dos pacientes a fim de monitorá-los de maneira ativa e mitigar riscos de incidentes comportamentais (p. ex., fuga, agressão — no caso de comorbidades de saúde mental/comportamental) ☐

3.1.5 Componentes da edificação

- Dispor de entradas/portas para pacientes que:
  - incluam painéis com visão para observação externa ☐
  - detenham esporos em aerossol ☐
  - permitam a passagem de equipamento e entrada e saída de macas ☐
- Permitir acabamentos laváveis e de fácil limpeza nas áreas de pacientes (avaliar a remoção de acabamentos existentes, como revestimentos de paredes, cortinas e carpete) ☐
- Proporcionar piso de superfície dura e antiderrapante ☐

3.1.6 Equipamentos e mobiliário

- Usar para pacientes leitos e cadeiras que possam ser desinfetados ☐
- Avaliar o espaço necessário para mobiliário e equipamentos médicos ☐
- Remover os equipamentos ou o mobiliário que não estejam sendo usados diretamente para o atendimento ao paciente ☐

3.1.7 Transporte

- Os elevadores devem permitir que o funcionário mova o paciente em maca ou, se for ambulatorial, em cadeira de rodas ☐
- Considerar a separação do fluxo de funcionários e pacientes nos elevadores ☐

3.2 Estruturais

- Confirmar se o sistema estrutural existente pode suportar a carga estrutural (ativa) revista para uma quantidade maior de ocupantes, equipamento médico e equipamento adicional no telhado ☐

3.3 Mecânicas

3.3.1 Isolamento e pressão negativa

- Se for clinicamente necessário, tomar providências para manter uma sala, zona ou andar de pressão negativa ☐
- Se possível ou clinicamente necessário, utilizar/criar salas de isolamento para infecções transmitidas por via aérea ☐
- Onde for possível, localizar os exaustores perto da cabeça do paciente ☐
- Garantir que os espaços adjacentes estejam protegidos contra contaminação cruzada ☐

3.3.2 Trocas de ar e filtração

- Onde for possível, a preferência são dez trocas de ar por hora, mais duas trocas de ar externo por hora ☐
- Onde for possível, a preferência é usar filtros MERV 7 nas áreas com pacientes e filtros finais MERV 14 ☐

3.3.3 Teste e ajuste

- Realizar testes após a construção/reforma e ajustar os sistemas AVAC ☐

3.3.4 Limitações potenciais dos sistemas existentes

- Considerar as unidades de parede e os exaustores de banheiro quanto à capacidade de exaustão, filtração e taxas de renovação de ar existentes (podem ser afetados pelo tamanho dos dutos) ☐

- Considerar unidades de telhado acondicionadas com aquecimento elétrico para fornecer ventilação adicional ☐
- Considerar melhorias nos sistemas de ventilação do edifício (p. ex., ventiladores de maior volume) para manter a pressão negativa ☐
- Avaliar os controles de umidade para oferecer níveis otimizados de umidade para atendimento e tratamento eficazes dos pacientes ☐
- Considerar unidades de recirculação com filtro HEPA nos quartos dos pacientes ☐

3.3.5 Controle de fumaça

- Confirmar a capacidade dos sistemas de controle de fumaça. Se não for possível, o número de pacientes deve ser limitado ☐

3.4 Elétricas

3.4.1 Eletricidade

- Oferecer circuitos elétricos com base na demanda de equipamentos em todos os recintos para pacientes e funcionários ☐
- Confirmar a localização dos quadros de distribuição de circuitos para a distribuição elétrica geral ☐

3.4.2 Disponibilidade de circuitos terminais

- Confirmar a disponibilidade de circuitos terminais para apoio ao atendimento de pacientes ☐

3.4.3 Eletricidade de emergência

- Com base nas necessidades dos pacientes (ou seja, tratamento ambulatorial ou crítico), disponibilizar circuitos elétricos críticos ininterruptos e de segurança vital para apoiar áreas de atendimento aos pacientes durante pelo menos 24 horas para serviços essenciais, inclusive:
  - equipamento de sustentação da vida e equipamentos/refrigeradores para armazenamento de medicamentos ☐
  - tomadas para equipamentos médicos em postos de tratamento de pacientes e nas cabeceiras ☐
  - sistema de chamada de enfermeiros ☐
  - TI (relativa aos pacientes, comunicação da equipe médica e infraestrutura) ☐
  - iluminação das saídas ☐
  - sistema de alarme de incêndio, elevadores, infraestrutura de TI, etc. ☐
  - elevadores ☐
  - chave de transferência automática ☐

3.4.4 Iluminação

- Disponibilizar iluminação (luminárias vedadas) e lâmpadas portáteis de exame para apoio aos procedimentos clínicos ☐
- Verificar a iluminação das saídas de emergência ☐

3.5 Encanamento/gás medicinal

3.5.1 Gás medicinal

- Disponibilizar acomodações para oxigênio, ar medicinal e vácuo em apoio aos procedimentos de entubação e ventilação para acomodar demanda alta ou plena ☐
- Confirmar a disponibilidade de espaço para geradores de tanques de oxigênio modulares temporários quando necessário ☐
- Confirmar a disponibilidade de recipientes ou salas adequados resistentes a incêndio para armazenamento de oxigênio engarrafado ☐
- Confirmar a disponibilidade para armazenamento de tanques de oxigênio vazios ☐

3.5.2 Água

- Disponibilizar água potável para apoio aos procedimentos clínicos (pode incluir desinfecção da tubulação principal e do sistema de distribuição de água, e testagem analítica da edificação para confirmar se o sistema de água é seguro para o atendimento a pacientes) ☐
- Caso necessário, planejar o processamento de água para descontaminação ou esterilização no local ☐
- Planejar o gerenciamento contínuo do sistema de água de modo a incluir descarga, monitoramento da temperatura e níveis residuais de desinfetantes ☐

3.6 Proteção contra incêndios/segurança vital

- Confirmar as condições e expectativas quanto a incêndios/segurança vital junto às autoridades com jurisdição local ☐
- Confirmar a presença de sistemas e funcionalidade de alarmes contra incêndio em cenários de atendimento a pacientes. Caso não haja nenhum sistema de alarme contra incêndio, reavaliar a adequação da edificação para uma resposta a pandemias, ou instalar um sistema de alarme contra incêndios ☐
- Preferência: sistema totalmente automático de supressão de fogo ☐
- Confirmar a disponibilidade e espaçamento adequados de extintores de incêndio ☐

3.7 Comunicação

3.7.1 Tecnologia da informação

- Confirmar ou instalar infraestrutura segura de tecnologia da informação adequada para apoio ao atendimento a pacientes, em conformidade com a HIPAA ☐

3.7.2 Comunicações para serviços médicos de emergência

- Confirmar ou instalar um sistema de comunicação adequado para serviços médicos de emergência em apoio ao atendimento a pacientes, em conformidade com a segurança da HIPAA ☐

3.7.3 Chamada/comunicação com enfermagem

- Confirmar ou instalar um sistema de chamada de enfermeiros adequado e seguro para suporte do atendimento a pacientes ou método semelhante de comunicação do paciente com a enfermagem ☐
- Estabelecer um método para a comunicação dos clínicos (p. ex., celular, sem fio) ☐

3.7.4 Alarme contra incêndio

- Confirmar ou instalar um alarme contra incêndio conectado aos serviços de emergência locais ☐
- Verificar a disponibilidade de estações com alarme manual contra incêndio ☐

3.7.5 Vigilância de segurança

- Avaliar as necessidades de segurança e disponibilizar um sistema de vigilância conforme for adequado (câmeras, monitores, detectores de movimento, alarme para portas e janelas, etc.) ☐
- Considerar sistemas sem fio quando necessário ☐

4.0 Considerações adicionais

4.1 Populações etnicamente diversas ou marginalizadas

- Rever as necessidades de espaço para atender à competência cultural ☐
- Considerar acomodações para práticas culturais e espirituais específicas ☐
- Considerar a acessibilidade do local alternativo de atendimento e opções de transporte dentro ou adjacentes a comunidades residenciais ☐
- Disponibilizar espaço adequado para assistentes sociais e serviços de interpretação de idiomas ☐

4.2 População rural

- Considerar a acessibilidade do local alternativo de atendimento e o transporte até ele, avaliando o tempo de deslocamento e a distância ☐
- Considerar a necessidade de transporte por helicóptero ☐
- Avaliar as necessidades de hospedagem e alojamento para profissionais de saúde e familiares dos pacientes ☐
- Avaliar a necessidade de transporte por ambulância de pacientes mais graves ☐
- Verificar os sistemas de comunicação (telefone celular, repetidores para uma cobertura melhor) ☐
- Verificar se as necessidades críticas de infraestrutura (saneamento, água potável, eletricidade) são adequadas à demanda prevista ☐

4.3 Populações em situação de rua ou desabrigadas

- Disponibilizar banheiros com chuveiros perto da entrada ☐
- Designar uma área de armazenamento para pertences, bicicletas, etc. ☐

4.4 Pacientes com problemas mentais e comportamentais

- Segundo a sessão 1.1.4, realizar uma avaliação de risco de segurança em todos os locais de acesso a pacientes com problemas mentais e comportamentais em potencial para eliminar a possibilidade de automutilação e danos contra terceiros ☐
- Caso pacientes com problemas mentais ou comportamentais sejam identificados, proporcionar espaço para observação individual contínua até sua transferência ou alta ☐
- Considerar quartos seguros, se necessário para o plano operacional identificado ☐

Diretrizes adicionais

Para materiais adicionais de referência, consulte https://bit.ly/C19HealthcareArch

Contribuições do grupo de trabalho e dos autores

Membros do grupo de trabalho

(MS) Molly M. Scanlon, PhD, FAIA, FACHA – Presidente
(DA) Diana Anderson, MD, ACHA
(EE) Eve A. Edelstein, PhD, MArch, Assoc. AIA, EDAC, FAAA
(JF) John Fowler, AIA, NCARB, EDAC, LEED AP
(WH) William Hercules, FAIA, FACHA, FACHE
(EP) Erin Peavey, AIA, EDAC, LEED AP
(YS) Yiselle Santos, AIA, LSSYB, WELL AP, LEED AP
(ET) Ellen Taylor, PhD, AIA, MBA, EDAC
(KW) Kirsten Waltz, AIA, ACHA, EDAC, LEED AP
(MW) Marvina Williams, RN, BSN, LSSBB
(FZ) Frank Zilm, DArch, FAIA, Emérito FACHA
(DP) Doug Paul, Equipe do AIA

Ferramenta de avaliação do estado de preparação de locais alternativos de atendimento para Covid-19
Os itens seguintes representam as contribuições principais do autor: conceptualização, MS; análise de códigos e padrões, JF, WH, KW e ET; administração de projetos, ET, MS e DP.

Reuniões
O grupo de trabalho realizou reuniões diárias de 24 de março a 2 de abril de 2020 para a preparação do Sumário #1 sobre o Impacto na Saúde e de trabalhos afins para revisão, consenso e aprovação. Foram realizadas reuniões de subgrupos adicionais durante o mesmo período de tempo. Atualizações foram preparadas até o dia 22 de abril de 2020 para o lançamento da versão revisada

Contribuidores adicionais para a Ferramenta de avaliação do estado de preparação de locais alternativos de atendimento para Covid-19
Amanda Abney, Associate AIA, EDAC
Patricia Acevedo, AIA
Javan Arroyo
Joe Bonanno
Gabrielle Bullock, FAIA, NOMAC, IIDA, LEED AP
Daniel Caron, PE, LEED AP
Jason Costello, AIA, EDAC
Ashleigh Cotter, CID, CHID, IIDA, LEED AP BD+C, CLGB
Rachael Farrell, EDAC, LSSGB, LEED AP, BD+C
David Frum, FAIA, FACHA
Ruth Fanning, MD
Angel Gomez
Jeff Hankin, PE
Anita Honkanen, MD
Nathan Howell, AIA
Jennifer Burke Jackson, AIA, LEED AP
Isaac Karley
Ed Ledoux
Christopher Lynch, PE
Zach Mathern
Don MacAlister, CHPA
Todd Medd, AIA, NCARB
Christopher O'Brien, AIA, ACHA, EDAC, LEED AP
Sam Olbekson, AIA, NCARB, AICAE
John P. Riordan, MD
Nick Schumacher, PE
Michael Sheerin, PE, LEED AP
Hannah Shultz
Tom Smith, CHPA, CPP
Rick Taylor, RCDD
Kevin Tuohey, CHPA
Scott Waltz, RA
Julie Wellik
Tony York, CHPA, CPP

The American Institute of Architects
(Instituto Americano de Arquitetos)
1735 New York Ave., NW
Washington, DC 20006
aia.org
